# Patient education booklet to support evidence-based low back pain care in primary care – a cluster randomized controlled trial

**DOI:** 10.1186/s12875-021-01529-2

**Published:** 2021-09-07

**Authors:** Anna S. Simula, Hazel J. Jenkins, Mark J. Hancock, Antti Malmivaara, Neill Booth, Jaro Karppinen

**Affiliations:** 1 Medical Research Center Oulu, Oulu University Hospital and University of Oulu, P.O. Box 8000, 90014 Oulu, Finland; 2 Department of General Medicine, the South Savo Social and Health Care Authority, Porrassalmenkatu 35-37, 50100 Mikkeli, Finland; 3 Department of Health Professions, Faculty of Medicine, Health, and Human Sciences, Macquarie University, Balaclava Road, North Ryde, NSW 2109 Australia; 4 Department of Chiropractic, Faculty of Medicine, Health, and Human Sciences, Macquarie University, Balaclava Road, North Ryde, NSW 2109 Australia; 5 Finnish Institute for Health and Welfare, P.O. Box 30, 00271 Helsinki, Finland; 6 Faculty of Social Sciences (Health Sciences), Tampere University, Arvo Ylpön katu 34, 33014 Tampere, Finland; 7 Finnish Institute of Occupational Health, Aapistie 1, 90220 Oulu, Finland; 8 Rehabilitation Services of South Karelia Social and Health Care District, Lappeenranta, Finland

**Keywords:** Low back pain, Primary care, Cluster randomized study, Guideline implementation, Patient education, Low back pain imaging

## Abstract

**Background:**

Inappropriate imaging and low-value care for low back pain (LBP) are common. A new patient-education booklet was created to overcome identified barriers to the delivery of recommended care, including the use of inappropriate imaging. Our aim was to assess the effectiveness of this booklet as part of primary care for LBP patients in comparison to usual care.

**Methods:**

A cluster-randomized trial was performed. The intervention involved providing practitioners with the new patient-education booklet and a 30-min training session on its use. The booklet was provided during the clinical consult to all consenting LBP patients in the intervention group. Primary outcomes were the proportion of patients presenting with LBP who underwent imaging examinations during the first three months of follow-up and PROMIS PF-20 (Patient-Reported Outcomes Measurement Information System, 20-item physical functioning short form) change between baseline and three-month follow-up. Secondary outcomes, including sick leave and imaging examinations at 12 months, were investigated. Logistic regression using GEE-estimation was used for dichotomous outcomes, Poisson regression using GEE-estimation for count outcomes, and linear mixed models for continuous outcomes.

**Results:**

Using the patient education booklet appeared to substantially reduce the proportion of LBP patients who underwent an imaging examination at three months, but the result was not statistically significant (OR 0.57, 95% confidence interval (Cl) 0.27 to 1.22). At 12 months, the effect was slightly larger and statistically significant (OR 0.50, 95%Cl 0.30 to 0.83, p = 0.008). No difference was observed in the PROMIS PF-20 T-score change between baseline and 3 months or 12 months (p = 0.365 and p = 0.923, respectively). The number of sick leave days in the intervention group was less than that in the control group at 3 months (RR 0.47, 95%Cl 0.26 to 0.83, p = 0.010) and at 12 months (RR 0.36, 95%Cl 0.18 to 0.72, p = 0.004).

**Conclusions:**

The booklet appeared to be effective in reducing the proportion of LBP patients who underwent imaging examinations over 12 months. The intervention had no discernible effect on the PROMIS PF20 T-score change. The number of sick leave days was substantially lower in the intervention group.

**Trial registration:**

ISRCTN, ISRCTN14389368, Registered 4 April 2019—Retrospectively registered.

**Supplementary Information:**

The online version contains supplementary material available at 10.1186/s12875-021-01529-2.

## Background

Low back pain (LBP) has been estimated as the leading cause of global years lived with disability in many countries and is one of the most costly health problems worldwide [1–3]. Management of LBP is often inconsistent with guidelines, and low-value care, such as inappropriate imaging, is common [4, 5]. For example, one third to one half of LBP patients undergo inappropriate imaging [6], which has been associated with increased health care costs, increased downstream health care utilization and increased disability [7–10]. Increased compliance with guidelines has shown to reduce health care costs and worker’s compensation claims [11].

The implementation of clinical guidelines for LBP is complicated and has several physician- and patient-reported barriers [12, 13]. Australian researchers have developed a new patient education booklet which focuses on both physician- and patient-related barriers and: 1) provides patient and practitioner education; 2) reminds practitioners of recommended care; 3) provides clinical decision support; and 4) facilitates practitioner-patient communication [14]. The booklet has been translated into and validated in Finnish. Preliminary evaluation by patients and practitioners in Finland has suggested that the booklet may be helpful in LBP management and in decreasing the need for LBP imaging [15].

### Trial objectives

The primary objective of this cluster randomized controlled trial was to assess the effectiveness of the “Understanding low back pain” patient-education booklet in addition to usual care in comparison to usual care alone, in reducing the proportion of patients presenting with LBP who undergo imaging examinations due to LBP over the first three months of follow-up (individual participant-level data), and to improve physical functioning at three-month follow-up (individual participant-level data).

The secondary objective was to assess the effectiveness of the intervention in reducing the proportion of patients presenting with LBP who undergo imaging examinations due to LBP, LBP-related sick leave days, health care appointments and disability, and to improve physical functioning and quality of life over 3- and 12-month follow-ups (individual participant-level data).

## Methods

We used a cluster-randomized trial design to assess the effectiveness of using the patient education booklet with LBP patients in primary care in comparison to usual care. Ethics approval was granted by the Ethics Committee of the University Hospital of Oulu. The study was reported in accordance with the CONSORT 2010 checklist with extension for Cluster Trials. The trial was retrospectively registered at ISRCTN (ISRCTN14389368, Registered 4 April 2019).

### Trial design and participants

**Fig. 1:**
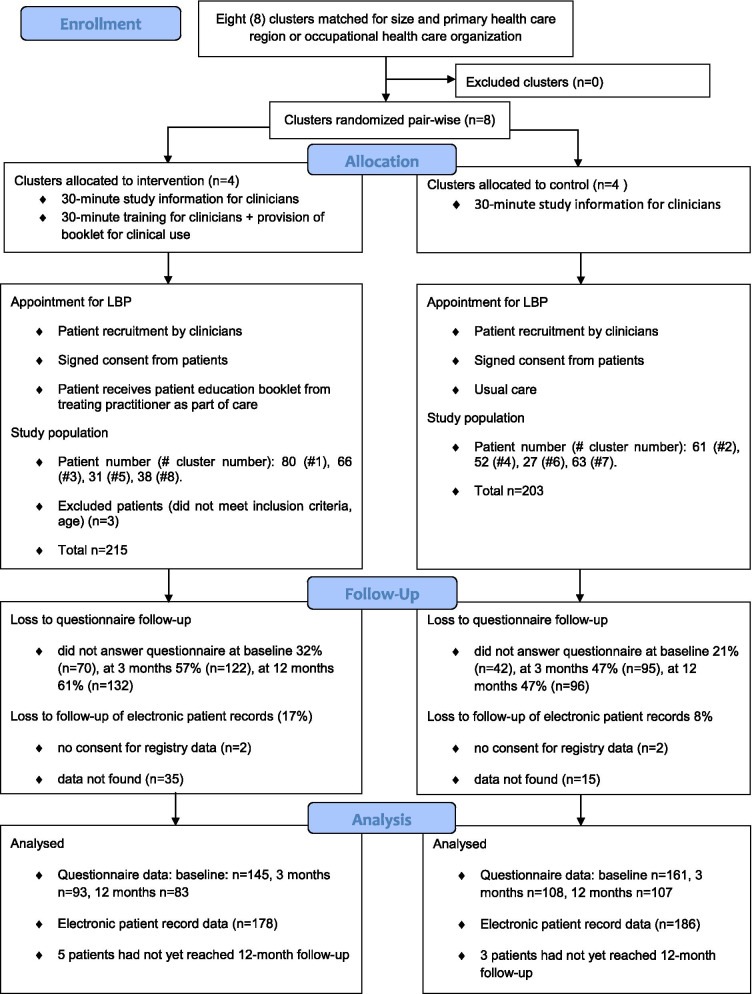
Flow chart

Figure 1 presents a flow chart of the trial design and participants. We used cluster randomization to minimize contamination between the intervention and control groups. Cluster randomization was performed at the health care region level rather than at the practitioner level, because patients would be evaluated by different health care professionals in the same health care region. We selected six public health care regions and two occupational health service organizations for clusters. The clusters were chosen and matched according to population size and type of health care provider (public or occupational) (Fig. 1). The managers of all the participating centres in the clusters were informed of the study and consented to participate before pairwise randomization into control or intervention groups was performed by an external statistician using a random number generator. Blinding was impossible because of the study design [16]. All the physicians and physiotherapists in the study clusters were invited to participate in the study and recruit eligible patients for the study.

In all the centres (in both the intervention and control groups), we provided the professionals (physiotherapists, nurses and physicians) with a 30-min training session, which covered the eligibility criteria and the overall study procedures (see Powerpoint slide show, Additional file 1). The physicians, physiotherapists and nurses identified eligible patients when they attended appointments for LBP. Selection bias was reduced by involving large numbers of professionals in the recruitment procedure. The professional explained the study to the patient and requested their consent. The professionals then sent the signed patient-consent forms to the research assistant (outside the centre). Whether a patient initially presented to a physiotherapist, physician or nurse did not change because of the study process. In Finland, LBP patients may initially present to a: 1) physician; 2) physiotherapist; 3) nurse together with a physician; or 4) nurse alone (the first contacted professional), depending on local care policies, the education level of the professionals, the availability of appointments at the time, and the patient’s symptoms (usually determined by the triage nurse over the phone). The first professional each patient contacted and patient baseline information was identified from electronic patient record data. The research assistant repeatedly sent information and a reminder about the study to the physicians, physiotherapists and nurses every month. The email contained a patient information sheet, brief information on patient recruitment, and details on eligibility criteria. We asked them, to recruit consecutive patients.

The patients who were eligible for inclusion were aged 18 to 65 and presented for treatment for LBP with or without associated radicular pain, that had lasted for two weeks or less (low risk of prolonged pain) for the first time. The exclusion criteria included a suspected serious cause of LBP (for example, cancer, fracture or infection); or LBP requiring urgent (emergency) care, such as cauda equina syndrome. Only patients who signed a consent form were included as participants in the study and patients were free to discontinue their participation in the study at any time without a reason.

### Intervention

The main focus of the intervention was the use of the patient education booklet in addition to usual care as part of the appointment with the professional (individual patient-level intervention). The professionals (physicians, physiotherapists and nurses) in the intervention arm attended a 30-min group training session on the booklet’s content and how it should be used during appointments (see Powerpoint slide show, Additional file 2) (cluster-level intervention). The training sessions were held during the weekly clinic meetings to enhance participation. Smaller units could alternatively participate online. After the education session, the professionals were encouraged to use the booklet (printed Finnish version) in the manner that they preferred (e.g., to guide the whole interaction with the patient, or to emphasize key points at the end of the consultation, etc.) during the LBP patients’ appointments. In the trial, patients were considered to have received the intervention if the health professional provided the booklet to the patient during or after the consultation, with or without further discussion. This was done in order to avoid potential limitations to the delivery of the intervention caused by health care professionals’ time limits or patients’ receptivity to education. Health care professionals were asked to recruit consecutive patients; however, records were not kept concerning whether or not consecutive patients who met the inclusion criteria were recruited by the health-care professionals. The English version of the booklet is available online (https://tinyurl.com/lowbackpaineducation). Other aspects of care were provided as usual. All the consenting LBP patients in the intervention health care units received the booklet from their physician, physiotherapist or nurse.

### Control

The professionals in the control arm received a short training session on the study and recruitment procedure only. All the consenting LBP patients presenting to the control clinics received usual care without the booklet.

### Outcomes

The two primary outcomes were as follows: 1) The proportion of patients presenting with LBP who underwent imaging examinations due to LBP during the first three months of follow-up; and 2) Change in PROMIS PF-20 T-score (Patient-Reported Outcomes Measurement Information System, 20-item physical functioning short form) from baseline to three-month follow-up (individual patient level) [17, 18]. Imaging data were collected from electronic patient record data to determine the proportion of patients presenting with LBP who had undergone imaging examinations due to LBP (yes/no). The patient-reported outcomes were collected using web-based questionnaires.

The secondary outcomes were determined from the electronic patient record data on the basis of the following: the proportion of patients presenting with LBP who had undergone imaging examinations due to LBP over 12 months of follow-up; imaging examinations stratified by the type of imaging [radiographs/ magnetic resonance imaging (MRI)/computer tomography (CT)] over 3- and 12-month follow-ups; the number of LBP-related sick leave days; and the number of LBP-related health care professional appointments (primary care: physician, physiotherapist, nurse; secondary health: physiatrist and orthopaedist) during the 3- and 12-month follow-up.

The secondary patient reported outcomes were: change in Oswestry Disability Index (ODI) between baseline and 12-month follow-up [19]; change in Roland Morris disability questionnaire between baseline and 12-month follow-up; change in PROMIS (Patient-Reported Outcomes Measurement Information System) (short form 20a) between baseline and 12-month follow-up; change in LBP intensity (0–10 Numerical Rating Scale (NRS)) during past week between baseline and 3- and 12-month follow-ups; change in leg pain intensity (0–10 NRS) during past week between baseline and 3- and 12-month follow-ups; and change in EQ-5D (EuroQol five dimensions) between baseline and 12-month follow-ups [20] and Frequency of LBP during last three months at baseline, 3- and 12-month follow-ups (daily LBP yes/no).

### Electronic patient record data

In each health care region or organization of the study, all the primary health care professionals (physicians, physiotherapist, nurses, etc.) use the same electronic patient record data. The data are organized according to professions (e.g., one file for primary health care physicians, another file for physiotherapists, etc.). Imaging examinations are documented on separate files and contain lists of completed examinations and imaging reports. All health care professionals have access to both primary and secondary electronic health care data directly or through passcode-protected access to the nationwide database. The input of health data is obligatory for health care providers. Nationwide electronic patient record data are accessible to all health care providers in Finland. The data were collected manually from the organizational database by the first author or one of two research assistants, using a paper checklist for each patient (Additional file 3). Initially, to ensure congruency, the checklist data for 10 patients were extracted independently by two people and compared. The original data collection sheets of each patient have been retained to allow subsequent data checks if required. Electronic patient record data were collected from the date of informed signed consent up to the 3- and 12- month follow-ups.

### Patient questionnaire data

Patient-reported data were collected using online questionnaires sent to the patients via an email hyperlink. The baseline questionnaire was collected after the patient consented to participate and after the first appointment; the follow-up questionnaire was collected 3 months and 12 months after the baseline questionnaire. If the patients did not complete the baseline and follow-up questionnaires within one week, the research assistant resent the hyperlink via email. If an email address was missing, the research assistant sent a paper version of the questionnaire. Further reminders were sent at two weeks and three weeks to the patients who had not responded to the baseline, 3-month or 12-month questionnaires by a text message containing a hyperlink to the related questionnaire.

### Changes to the protocol

Changes were made to the original protocol because of unreliable data relating to one of the original secondary outcome measures (pain medication) and in order to respect the original purpose of the STarT Back Tool (SBT). We did not include the secondary outcome of pain medication as we could not accurately extract these data from the electronic records, and we only used SBT to describe baseline differences, not as a secondary outcome measure.

### Sample size

At baseline, the imaging rate (radiographs, MRI or CT) was expected to be 30%. We used a web-based calculator to compare the proportion with a dichotomous outcome (imaging/nor imaging) of the two samples (http://www.sample-size.net/sample-size-proportions/). We estimated the Design Effect (DE) for unequal clusters in a cluster-randomized design to increase the statistical power of the cluster randomized study: DEunequal = 1 + [(1 + CV^2^)x m -1]ρ; when ρ(ICC) = 0.01; coefficient of variation of cluster size (CV) was 0.208; CV = sd/m, (sd = standard deviation of mean cluster size; m = mean cluster size); sd = CSrange/4 (CS = cluster size) [21]. A sample size of 408 patients would enable the detection of an absolute 15% decrease in imaging proportion with 80% power and a type I error of 0.05.

The sample of 408 could provide more than adequate power for the other primary outcome of PROMIS based on the sample size a web-based calculator for Inference for Means in clustered samples (http://www.sample-size.net/means-sample-sizeclustered/). The Minimal Important Difference (MID) for change in PROMIS PF-20 was about two points, and standard deviation (SD) was 3.66 [22]. The type I error rate was 0.05, and the Intracluster Correlation Coefficient (ICC) 0.025 [23]. A sample size of 128 patients would enable the detection of a difference of two points in PROMIS PF-20 with 80% power.

### Statistical methods

We analysed the between-group differences at baseline using independent-samples t-tests for normally distributed continuous variables, the Mann–Whitney U-tests for non-normally distributed continuous variables, and the Chi-square test or Fisher’s exact tests for dichotomous variables.

The statistical methods used to analyse the differences between the intervention and control groups were selected according to the type of the outcome. We used logistic regression for dichotomous outcomes (imaging and LBP frequency), providing an estimated difference between the groups expressed as odds ratio (OR) with a 95% confidence interval (CI). Poisson regression was used for count outcomes (health care professional appointments and sick leave days), providing an estimated difference between the groups expressed as risk ratio (RR) with a 95% confidence interval. Logistic and Poisson models were analysed using generalized estimating equations (GEE) with an exchangeable working correlation to account for the clustered nature of the data. We analysed the continuous outcomes (PROMIS, ODI, NRS for pain, EQ-5D index and EQ VAS) using a linear mixed model with random effects for clusters, providing an estimated least squares mean difference with a 95% confidence interval. Age-adjusted analyses were also conducted for all outcomes because of baseline differences.

Post-hoc sensitivity analyses were conducted for the primary outcomes based on the first contacted professional, as this was unbalanced between the groups and there was strong rationale for why the intervention may be more or less effective depending on the profession of the first professional contacted. In the Finnish health care system, physiotherapists cannot directly refer patients for imaging, so which professional the patient first contacted was particularly important for our imaging outcome. Statistical analyses were conducted using IBM SPSS Statistics for Windows, version 26 (IBM Corp., Armonk, NY). Two-sided tests were used, and p-values less than 0.05 were considered statistically significant.

## Results

### Baseline characteristics

The number of professionals who attended the training and study information sessions and participated in recruiting the intervention group were: Cluster 1: 18 of the 25 physicians, all 8 physiotherapists and 16 nurses; Cluster 3: 20 of the 31 physicians and 8 of the 16 physiotherapists; Cluster 5: 14 of the 19 physicians and 5 of the 8 physiotherapists; cluster 8: 7 of the 10 physicians and 1 of the 5 physiotherapists.

The number of professionals who attended the study information sessions and participated in recruiting the control group were: Cluster 2: all 46 physicians and 12 of the 14 physiotherapists, 37 nurses; Cluster 4: 17 of the 27 physicians and 5 of the 12 physiotherapists; Cluster 6: 7 of the 25 physicians and 5 of the 6 physiotherapists; Cluster 7: 13 of the 18 physicians and 7 of the 13 physiotherapists.

In total, 418 LBP patients consented to participate in the study. Recruitment was carried out between 13^th^ April 2017 and 30^th^ May 2020. The number of participants in each cluster, the missing data in the follow-up questionnaires, and the missing data in the electronic patient records data are shown in Fig. 1. The patients in the intervention and control groups had similar characteristics at baseline (Tables 1 and 2). Approximately half of the patients were actively working. The patients’ mean age was 41.4 years in the intervention group and 44.6 years in the control group (p = 0.011). No differences were found between the PROMIS T-score of the intervention and control groups at baseline. However, the first contacted professional who used the booklet during the appointment differed significantly (p = 0.001; Table 2). In the intervention group, the first contacted professional was a physician for 44% and a physiotherapist for 43% of the patients, in comparison to 28% and 62% in the control group, respectively. Only a few patients met both a physician and a nurse (12% in the intervention and 9% in the control group). The first contacting professional was seldom a nurse alone (0% in the intervention and 1% in the control group).

**Table 1:** Baseline characteristics of study population

Characteristics	Intervention (n = 212)	Control (n = 203)	missing %(n)	P value for difference between intervention and control groups
Age^a^ (years)	41.4 (12.8)	44.6 (12.6)	0 (0)	**0.011**
Gender female^b^	60.8 (129)	67.0 (136)	0 (0)	0.193
Physically inactive^b^ (light exercise ≤ 1/month)	4.8 (7)	3.7 (6)	26.3 (109)	0.778
Body mass index^a^ (kg/m^2^)	27.8 (5.8)	27.8 (5.2)	26.3 (109)	0.984
Smoking^b^	29.0 (42)	29.8 (48)	26.3 (109)	0.871
DEPS score^c^	5.0 (8.0)	6.0 (8.5)	26.3 (109)	0.118
Actively working^b^	57.9 (84)	56.5 (91)	26.3 (109)	0.804
Work ability^c^ (0–10)	7.0 (8.0)	7.0 (8.0)	26.3 (109)	0.279
Comorbidity ^b^			26.9 (112)	
*Diabetes*	3.5 (5)	9.3 (15)		0.062
*Rheumatoid arthritis*	1.4 (2)	1.9 (3)		1.000
*Spondylarthritis*	0	0.6 (1)		1.000
*Osteoarthritis*	18.2 (26)	25.5 (41)		0.130
*Depression*	17.5 (25)	22.4 (36)		0.317
*Fibromyalgia*	2.1 (3)	3.1 (5)		0.727
*Inflammatory bowel disease*	1.4 (2)	5.0 (8)		0.110
*Muscle disease*	0.7 (1)	0		0.470

^a^Mean (standard deviation), p value for between-group difference from independent-samples T-test

^b^Percentage (frequency), p value for between-group difference from Chi-square test or Fisher’s exact

^c^Median (interquartile range), p value for between-group difference from Mann–Whitney U-test

LBP (Low Back Pain), DEPS (Depression Scale)

**Table 2:** Pain-related characteristics of the study population and first contacting professional during the study

Characteristics	Intervention (n = 212)	Control (n = 203)	P-value for the difference between intervention and control groups	Missing^^ in intervention %(n)	Missing^^ in control %(n)
**Electronic patient records data**					
First contacting professional^ % (n)^2^			**0.001**	10.8 (23)	9.4(19)
Physician	44.4 (84)	27.7 (51)			
Nurse and physician	12.2 (23)	9.2 (17)			
Physiotherapist	43.4 (83)	62.0 (114)			
Nurse alone	0.0 (0)	1.1 (2)			
**Baseline questionnaire data**					
Back pain intensity during past week^1^ (NRS, 0–10)	5.1 (2.4)	4.9 (2.5)	0.455	31.6 (67)	20.7 (42)
Leg pain intensity during past week^1^ (NRS 0–10)	3.7(3.3)	3.3 (3.0)	0.376	31.6 (67)	20.7 (42)
SBT total score^1^	4.5 (2.3)	4.6 (2.3)	0.702	31.6 (67)	20.7 (42)
SBT risk groups^2^			0.731	31.6 (67)	20.7 (42)
*Low risk*^*2*^	37.2 (54)	32.9 (53)			
*Medium*^*2*^	40.0 (50)	42.9 (69)			
*High*^*2*^	22.8 (33)	24.2 (39)			
ÖMPSQ-short total score^1^	43.0 (17.3)	43.2 (19.6)	0.914	31.6 (67)	20.7 (42)
ÖMPSQ-short risk groups*^2^			0.615	31.6 (67)	20.7 (42)
*Low risk*^*2*^	48.3 (70)	44.1 (71)			
*Medium*^*2*^	18.6 (27)	17.4 (28)			
*High*^*2*^	33.1 (48)	38.5 (62)			
Pain-related fear (FABQ)^3^	34.0 (29.5)	37.0 (36.5)	0.249	31.6 (67)	20.7 (42)
Pain-related fear (FABQ) -Work**^3^	13.0 (17.0)	16.0 (21.5)	0.213		
Pain-related fear (FABQ) -Physical activity***^3^	13.0 (8.0)	14.0 (9.5)	0.773		
Pain Self-Efficacy beliefs Questionnaire (PSEQ)^3^	44.0 (18.0)	45.0 (20.0)	0.908	31.6 (67)	20.7 (42)
Physical functioning (PROMIS PF-20 T-score)^1^	44.7 (6.7)	43.8 (7.4)	0.271	34.3 (73)	25.1 (51)
Physical impairment, (RMDQ)^3^	5.0 (7.0)	6.0 (8.0)	**0.049**	37.3 (79)	29.6 (60)
Disability (ODI%)^1^	23.9 (8.4)	24.1 (8.9)	0.832	31.6 (67)	20.7 (42)
Self-rated health status^1^ (1–100)	67.1 (21.1)	67.2 (20.8)	0.953	31.9 (68)	20.7 (42)
LBP frequence (daily LBP yes/no)^2^	43.4 (63)	50.9 (82)	0.191	31.6 (67)	20.7 (42)
Duration of LBP^2^			0.586	31.6 (67)	20.7 (42)
*3 weeks or less*	31.7 (46)	33.5 (54)			
*4 to 52 weeks*	44.8 (65)	47.8 (77)			
*over one year*	23.4 (35)	18.6 (30)			
Pain medication use ≥ 3 days during past week^2^	46.9 (68)	46.0 (74)	0.870	31.6 (67)	20.7 (42)

^1^Mean (standard deviation), p-value for between-group difference from independent-samples T-test

^2^Percentage (frequency), p-value for between-group difference from Chi-square test

^3^Median (interquartile range), p-value for between-group difference from Mann–Whitney U-test

^Contacts to other professional before and after study consent allowed

^^Missing data include lost to follow-up

^*^Low-risk (0‒39 points), Medium-risk (40‒49 points) and High-risk (50‒100 points)

^**^FABQ (Fear Avoidance Believes Questionnaire) work – items 6, 7, 9, 10, 11, 12, 15

^***^FABQ physical activity – items 2, 3, 4, 5

NRS (Numeral Rating Scale), SBT (Start-Back Tool), ÖMPSQ (Örebro Musculoskeletal Pain Screening Questionnaire), PSEQ (Pain Self Efficacy Questionnaire), PROMIS PS-20 T-score (Patient-Reported Outcomes Measurement Information System, 20-item physical functioning short form T-score), RMDQ-24 (Roland Morris Disability Questionnaire 24 form), ODI (Oswestry Disability Index)

^**^ and *** Waddell G, Newton M, Henderson I, Somerville D, Main CJ. A Fear-Avoidance Beliefs Questionnaire (FABQ) and the role of fear-avoidance beliefs in chronic low back pain and disability. Pain. 1993 Feb;52(2):157–68

### Intervention results

#### Primary outcomes

The use of the patient education booklet appeared to substantially reduce the proportion of LBP patients who underwent an imaging examination at three months, but the result was not statistically significant (OR 0.57, 95% confidence intervals (Cl) 0.27 to 1.22, p = 0.147) (Table 3). The proportion of patients presenting with LBP who had undergone imaging examinations due to LBP over three months was relatively low in both groups: 10.8% in the intervention and 16.7% in the control group.

**Table 3:** Outcomes from electronic patient record data

	**Intervention group n = 178**	**Control group n = 186**	**intervention vs control**	**Age adjusted intervention vs control**
	**%(n)**	**%(n)**	**OR (95% CI); P value**	**OR (95% CI); P value**
Imaging^a^ (yes/no)				
*3 months*	10.8 (19)	16.7 (31)	0.57 (0.27 to 1.22); 0.147	0.60 (0.29–1.24); 0.166
*12 months*	17.5 (31)	29.7 (54)	**0.50 (0.30 to 0.83); 0.008**	**0.52 (0.32 to 0.84); 0.007**
Radiographs^a^				
*3 months*	5.6 (10)	9.1 (17)	0.57 (0.25 to 1.29); 0.178	0.58 (0.25–1.33); 0.199
*12 months*	8.4 (15)	14.7 (27)	**0.55 (0.34 to 0.88); 0.013**	**0.58 (0.37 to 0.90); 0.016**
Magnetic resonance imaging (MRI) ^a^				
*3 months*	7.3 (13)	8.6 (16)	0.86 (0.49 to 1.52); 0.610	0.90 (0.55–1.48); 0.688
*12 months*	12.8 (23)	21.1 (39)	**0.61 (0.44 to 0.84); 0.002**	**0.64 (0.49 to 0.84); 0.001**
Computed tomography (CT) ^a^				
*3 months*	0 (0)	1.6 (3)	^c^	^c^
*12 months*	0.6 (1)	2.2 (4)	^c^	^c^
MRI + CT^a^				
*3 months*	7.3 (13)	9.1 (17)	0.81 (0.45 to 1.46);0.475	0.86 (0.51–1.4); 0.555
*12 months*	13.4 (24)	21.7 (40)	**0.60 (0.39 to 0.91); 0.016**	**0.64 (0.45 to 0.91); 0.014**
	**mean(SD); sum**	**mean(SD); sum**	**RR (95% CI); P-value**	**RR (95% CI); P-value**
Sick leave days^b^				
*3 months*	3.8 (11.2); 664	8.4 (20.2); 1526	**0.47 (0.26 to 0.83); 0.010**	**0.48 (0.29 to 0.81); 0.006**
*12 months*	7.5 (31.3); 1330	20.8 (59.2); 3717	**0.36 (0.18 to 0.72); 0.004**	**0.39 (0.22–0.70); 0.002**
Physician appointments ^b^				
*3 months*	1.1 (1.4); 156	1.2 (1.5); 188	0.92 (0.44 to 1.9); 0.813	0.92 (0.44 to 1.9); 0.822
*12 months*	1.6 (2.1); 231	1.8 (2.3); 283	0.87 (0.4 to 1.9); 0.725	0.87 (0.41 to 1.78); 0.678
Physiotherapist appointments^b^				
*3 months*	1.2 (1.7); 212	1.3 (1.1); 235	0.88 (0.56 to 1.40); 0.601	0.89 (0.55 to 1.43); 0.618
*12 months*	1.7 (2.6); 297	2.0 (2.2); 359	0.83 (0.55 to 1.2); 0.356	0.83 (0.56 to 1.23); 0.829
Nurse appointments^b^				
*3 months*	0.3 (1.2); 60	0.2 (0.7); 41	1.3 (0.20 to 8.42); 0.772	1.3 (0.20 to 9.69); 0.749
*12 months*	0.6 (1.8); 107	0.3 (1.0); 60	1.72 (0.34 to 8.66); 0.514	1.93 (0.34 to 11.04); 0.458
Secondary health care appointments (physiatrist + orthopedist)^b^				
*3 months*	0.3 (1.0); 48	0.3 (0.7); 55	1.06 (0.68 to 1.65); 0.801	1.10 (0.73 to 1.64); 0.662
*12 months*	0.6 (1.6); 87	0.7 (1.8); 132	0.81 (0.49 to 1.35); 0.426	0.84 (0.53 to 1.33); 0.447

^a^Difference between intervention and control groups analysed with logistic regression using generailzed estimating equations with exchangeable working correlation matrix

^b^Difference between intervention and control groups analysed with Poisson regression using general estimating equations with exchangeable working correlation matrix

Odds Ratio (OR), Confidence Interval (CI), Risk Ratio (RR)

^c^OR not possible due to zero frequency

The between-group difference in PROMIS PF-20 T-score change between baseline and three-month follow-up was not statistically significant (mean difference 1.0, 95% CI -1.5 to 3.5, p = 0.365) (Table 4). The intraclass correlation of PROMIS t-score change at three months was 0.00005.

**Table 4:** Patient reported outcomes

Outcome	Time	Intervention group n (total n = 145)	Control group n (total n = 161)	Intervention group Mean^a^ (SE)	Control group Mean^a^ (SE)	Mean difference^b^ (95% CI)	P value	Age-adjusted mean difference^b^ (95% CI)	P value
PROMIS T-score^b^	Baseline	139	152	44.7 (0.6)	43.8 (0.6)	0.9 (-0.7 to 2.6)	0.271	0.5 (-1.1 to 2.1)	0.526
	Change 3 m	84	95	2.5 (0.7)	1.5 (0.7)	1.0 (-1.5 to 3.5)	0.365	0.93 (-1.0 to 2.9)	0.346
	Change 12 m	67	91	2.2 (0.9)	2.3 (0.7)	-0.1 (-2.3 to 2.1)	0.923	0.09 (-2.3 to 2.2)	0.935
Low back pain intensity during last week (NRS, 0–10) ^b^	Baseline	145	161	5.1 (0.3)	4.8 (0.3)	0.29 (-0.8 to 1.3)	0.521	0.38 (-0.7 to 1.5)	0.427
	Change 3 m	91	105	-0.6 (0.5)	-0.4 (0.5)	-0.3 (-2.1 to 1.5)	0.732	-0.3 (-2.1 to 1.5)	0.695
	Change 12 m	79	105	-1.8 (0.5)	-1.3 (0.5)	-0.47 (-2.1 to 1.2)	0.517	-0.5 (-2.2 to 1.1)	0.462
Leg pain intensity during last week LN [(NRS 0–10) + 0.5]^e^ ^b^	Baseline	145	161	0.9 (0.1)	0.9 (0.1)	0.1 (-0.4 to 0.5)	0.805	0.1 (-0.4 to 0.6)	0.708
	Change 3 m	91	105	-0.2 (0.2)	0.01 (0.2)	-0.2 (-0.8 to 0.4)	0.407	-0.2 (-0.8 to 0.4)	0.395
	Change 12 m	79	105	0.4 (0.2)	-0.2 (0.1)	-0.1 (-0.7 to 0.4)	0.506	-0.2 (-0.7 to 0.4)	0.491
ODI%^b^	Baseline	145	161	23.5 (1.3)	24.0 (1.2)	-0.5 (-4.0 to 3.1)	0.795	0.2 (-3.4 to 3.7)	0.925
	Change 3 m	91	105	-5.1 (1.8)	-3.4 (1.7)	-1.7 (-7.6 to 4.1)	0.501	-1.7 (-7.6 to 4.1)	0.511
	Change 12 m	79	104	-5.7 (2.4)	-4.1 (2.2)	-1.6 (-10.1 to 6.9)	0.645	-1.7 (-10.2 to 6.7)	0.619
EQ5D-3L index^db^	Baseline	145	161	0.659 (0.023)	0.664 (0.022)	-0.005 (-0.089 to 0.080)	0.890	< 0.001 (< 0.001 to 0.084)	0.807
	Change 12 m	79	107	0.048 (0.029)	0.053 (0.025)	0.005 (-0.081 to 0.071)	0.894	-0.007 (-0.083 to 0.070)	0.859
Self-rated health EQ5D-VAS (0–100)^b^	Baseline	144	159	68.2 (2.5)	67.1 (2.4)	1.1 (-7.3 to 9.4)	0.771	0.5 (-7.9 to 8.8)	0.899
	Change 12 m	75	103	5.4 (2.4)	5.6 (2.1)	-0.1 (-6.5 to 6.2)	0–970	0.03 (-6.4 to 6.4)	0.992
				**% (n)**	**% (n)**	**OR (95% CI)**		**OR (95% CI)**	
LBP frequency (daily LBP yes/no)^c^	Baseline	147	161	43.4 (63)	50.9 (82)	0.79 (0.57 to 1.09)	0.143	0.80 (0.55 to 1.16);	0.244
	3 months			38.7 (36)	42.6 (46)	0.94 (0.61 to 1.44)	0.768	0.94 (0.50 to 1.49)	0.792
	12 months	83	107	28.9 (24)	32.7 (35)	0.85 (0.48 to 1.51)	0.586	0.84 (0.48 to 1.49)	0.560

Presented as intervention group vs control group. ^a^Values estimated from least square means with standard error. ^b^Least square mean difference estimated from linear mixed model with random effects for unit. Positive and negative mean differences indicate higher and lower values among intervention group, respectively. ^c^Difference between intervention and control groups analyzed with logistic regression using generalized estimating equations with exchangeable working correlation matrix

Change 3 m calculated from baseline to 3-month follow-up and change 12 m from baseline to 12-month follow-up

^d^UK TTO

NRS (Numeral rating scale), ODI (Oswestry Disability Index), PROMIS T-score (Patient-Reported Outcomes Measurement Information System, 20-item physical functioning short form T-score), EQ5D-3L index (EuroQol five dimensions, 3-level version)

^e^LN (natural logarithmic) transformation for Leg pain intensity used because of positively skewed distribution

#### Secondary outcomes

The proportion of patients who underwent any imaging over the 12-month follow-up was lower in the intervention group. The imaging rate over 12 months was 17.5% in the intervention and 29.7% in the control group (OR 0.50, 95% Cl 0.30 to 0.83, p = 0.008). Similar results were found when radiographs and MRI were analysed separately (at 12 months radiographs (OR 0.55, 95% Cl 0.34 to 0.88, p = 0.013) and MRI (OR 0.61, 95% Cl 0.44 to 0.84, p = 0.002)). The results were similar in the age-adjusted analyses (Table 3).

The mean number of sick leave days was 3.8 days in the intervention group and 8.4 days in the control group for the first three months, and 7.5 and 20.8 days, respectively, for the whole 12-month period. The differences in sick leave days were statistically significant over the 3-month (RR 0.47, 95% CI 0.26 to 0.83, p = 0.010) and 12-month follow-ups (RR 0.36, 95% Cl 0.18 to 0.72, p = 0.004; Table 3).

There were no statistically significant differences between the groups in the number of health care appointments (Table 3) or patient-reported secondary outcomes: physical functioning (PROMIS T-score), back or leg pain intensity, disability (ODI), quality of life (EQ-5D), self-rated health (EQ VAS) or frequency of daily LBP (Table 4).

### Sensitivity analyses

Sensitivity analyses were conducted on the basis of the first contacted professional (e.g., physician or physiotherapist), because of the large baseline differences and the likelihood that the effects of the intervention would be different across the different professionals (Table 5). When the first contacted professional was a physician, 7% of the patients underwent imaging examinations over the first three months in the intervention group, in comparison with 29% in the control group (OR 0.15, 95% CI 0.09 to 0.24, *p* < 0.001). Over 12 months, the proportion of imaging use was 15.7% in the intervention group and 42% in the control group (OR 0.23, 95% CI 0.16 to 0.33, *p* < 0.001). The intervention had no effect on PROMIS PF-20 change between baseline and 3-month or 12-month follow-up. In addition, when the first contacted professional was a physician, the intervention reduced sick leave days to one third at both 3 months (RR 0.35, 95% Cl 0.20 to 0.64, p = 0.001) and 12 months (RR 0.34, 95% Cl 0.23 to 0.51, *p* < 0.001). The risk of further physician appointments decreased by one third at both 3 months (RR 0.65, 95% Cl 0.55 to 0.75, *p* < 0.001) and 12 months (RR 0.60, 95% Cl 0.49 to 0.72, *p* < 0.001). The number of secondary health care appointments was lower at both 3 months (RR 0.79, 95% Cl 0.75 to 0.82, *p* < 0.001) and 12 months (RR 0.91, 95% Cl 0.88 to 0.95, *p* < 0.001).

**Table 5:** Sensitivity analyses according to first contacted professional

First contacted professional	Physician (Intervention n = 84, control n = 51)			Physiotherapist (Intervention n = 67, control n = 114)		
	**Intervention**	**Control**		**Intervention**	**Control**	
**Imaging (yes/no)**^**a**^	**% (n)**	**% (n)**	**OR (95% CI); P value**	**% (n)**	**% (n)**	**OR (95% CI); P value**
Imaging 3 months	7.3 (6)	29.4 (15)	**0.15 (0.09–0.24); < 0.001**	10.4 (7)	11.4 (13)	1.23 (0.30–5.61); 0.729
Imaging 12 months	15.7 (13)	42 (21)	**0.23 (0.16–0.33); < 0.001**	17.9 (12)	22.3 (25)	0.91 (0.37–2.25); 0.842
*Radiographs 3 months*	6.0 (5)	19.6 (10)	**0.21 (0.19–0.23); < 0.001**	4.5 (3)	6.1 (7)	0.88 (0.17–4.57); 0.882
*Radiographs 12 months*	8.3 (7)	23.5 (12)	**0.28 (0.25–0.31); < 0.001**	9.0 (6)	12.4 (14)	0.79 (0.31–2.02); 0.616
*Magnetic resonance imaging* (*MRI) 3 months*	3.6 (3)	13.7 (7)	**0.17 (0.09–0.32); < 0.001**	8.7 (6)	5.3 (6)	1.76 (0.57–5.48); 0.329
*MRI 12 months*	9.5 (8)	23.5 (12)	**0.33 (0.16–0.70); 0.004**	15.9 (11)	16.8 (19)	0.91 (0.48–1.71); 0.760
**PROMIS T-score**^**b**^	**Mean (SE)**	**Mean (SE)**	**Mean difference (CI); P value**	**Mean (SE)**	**Mean (SE)**	**Mean difference (CI); P value**
*Baseline*	42.8 (1.0)	42.2 (1.2)	0.60 (-3.9 to 5.1); 0.713	45.9 (0.9)	44.9 (0.8)	1.1 (-1.4 to 3.5); 0.391
*Change 3 m*	3.1 (1.1)	1.3 (1.2)	1.87 (-1.4 to 5.1); 0.254	2.2 (1.0)	1.3 (0.8)	0.9 (-1.7 to 3.6); 0.488
*Change 12 m*	2.7 (1.7)	1.3 (1.8)	1.44 (-5.0 to 7.9); 0.591	1.7 (1.7)	2.6 (1.3)	-0.9 (-8.7 to 6.8); 0.704
**Sick leave days**^**c**^	**Mean (SD)**	**Mean (SD)**	**RR (95% CI); P value**	**Mean (SD)**	**Mean (SD)**	**RR (95% CI); P value**
3 months	4.9 (10.6)	13.0 (23.2)	**0.35 (0.20–0.64); 0.001**	1.4 (7.8)	7.7 (21.6)	**0.24 (0.09–0.71); 0.010**
12 months	6.9 (16.7)	16.0 (30.1)	**0.34 (0.23–0.51); < 0.001**	4.5 (17.9)	28.6 (78.6)	**0.17 (0.07–0.45); < 0.001**
**LBP-related health care appointments**^**c**^						
Physician 3 months	1.6 (1.5)	2.2 (1.4)	**0.65 (0.55–0.75); < 0.001**	0.3 (0.6)	0.6 (1.2)	**0.39 (0.18–0.84); 0.017**
Physician 12 months	2.0 (2.2)	3.0 (2.0)	**0.60 (0.49–0.72); < 0.001**	0.9 (1.7)	1.2 (2.3)	0.72 (0.27–1.89); 0.501
Physiotherapist 3 months	0.8 (1.3)	0.6 (0.93)	0.90 (0.59–1.58); 0.897	2.0 (1.2)	1.6 (0.9)	**1.29 (1.16–1.43); < 0.001**
Physiotherapist 12 months	1.3 (2.6)	1.2 (1.6)	0.80 (0.47–1.37); 0.417	2.8 (2.1)	2.4 (2.6)	**1.05 (1.02–1.09); < 0.001**
Nurse 3 months	0.3 (0.8)	0.1 (0.4)	1.22 (0.22–6.81); 0.823	0.1 (0.3)	0.1 (0.3)	1.39 (0.39–4.99); 0.617
Nurse 12 months	0.8 (1.7)	0.2 (0.7)	1.73 (0.39–7.77); 0.474	0.3 (1.5)	0.1 (0.3)	**5.19 (1.16–23.3); 0.031**
Secondary health care 3 months	0.37 (0.92)	0.39 (0.67)	**0.79 (0.75–0.82); < 0.001**	0.2 (0.9)	0.3 (0.6)	0.86 (0.30–2.50); 0.783
Secondary health care 12 months	0.57 (1.17)	0.53 (0.98)	**0.91 (0.88–0.95); < 0.001**	0.6 (1.4)	0.8 (2.1)	0.84 (0.34–2.08); 0.699

Presented as intervention group vs control group.^a^Difference between intervention and control groups analysed with logistic regression using generalized estimating equations with exchangeable working correlation matrix. ^b^Least square mean difference estimated from linear mixed model with random effects for unit. Positive and negative mean differences indicate higher and lower values among intervention group, respectively. ^c^Difference between intervention and control groups analysed with Poisson regression using general estimating equations with exchangeable working correlation matrix

When the first contacted professional was a physiotherapist, the differences in imaging rates were not significant (3 months OR 1.23, 95% CI 0.30 to 5.61, p = 0.729; 12 months OR 0.91, 95% CI 0.37 to 2.25, p = 0.842). The PROMIS PF-20 T-score differed significantly at baseline when the first contacted professional was a physiotherapist, but the intervention was no more effective than usual care at 3-month or 12-month follow-up. However, the number of sick leave days decreased to one quarter in the intervention group at 3 months (RR 0.24, 95% Cl 0.09–0.71, p = 0.010) and to one fifth at 12 months (RR 0.17, 95% Cl 0.07 to 0.45, *p* < 0.001). Similarly, if the physiotherapists used the booklet as part of care, physician appointments decreased at three months (RR 0.39, 95% Cl 0.18 to 0.84, p = 0.017). At 12 months, the difference was smaller and no longer statistically significant (RR 0.72, 95% Cl 0.27 to 1.89, p = 0.501). There were no between-group differences in other secondary outcomes among the patients who first saw a physiotherapist.

The combination of a physician and nurse as the first contacted professional was uncommon (11% (n = 23) in the intervention group and 8% (n = 17) in the control group), and occurred in only two of the eight clusters. The sensitivity analyses of the effect of using the patient education booklet during an LBP patient appointment within this subgroup is presented in Additional file 4.

## Discussion

### Key findings

The use of the patient education booklet to support evidence-based care of LBP patients in primary care appeared to substantially reduce the proportion of imaging at three months; however, this was only statistically significant when a physician was the first contacted professional. The impact of using the education booklet on imaging rates was substantial at 12 months, with a reduction in imaging proportions of 50% in the intervention group compared to the control group. In the other primary outcome of change in PROMIS T-score, we found no difference between the intervention and control groups. In the secondary outcome of sick leave days, we observed a significant reduction both at 3 months (RR 0.47 95% CI 0.26 to 0.83, p = 0.010) and 12 months (RR 0.36 95% CI 0.18 to 0.72, p = 0.004).

Radiographic imaging rates were low in both the intervention and control groups (radiograph rate 8% in the intervention and 15% in the control group, MRI rate 13% and 21%, respectively) compared to a previous systematic review, in which 31% of patients underwent radiograph imaging due to LBP in primary care over one year, whereas 16% received MRI [24]. This difference may be explained by the profession of the first contacted professional. When the first contacted professional was a physician, the radiographic imaging rate over 12 months was 8% in the intervention group and 24% in the control group. In contrast, when physiotherapists were the first contacted professional, radiograph imaging use was 9% in the intervention group and 12% in the control group. In the Finnish health care system, physiotherapists cannot directly refer patients for imaging, which likely explains the lower imaging proportions and the stronger reduction in imaging in the sub-group of patients who contacted a physician first (OR 0.15, 95% CI 0.09 to 0.24). In addition, also the probability of severity of disease might differ according to the first contacting professional, which we are unable to evaluate in our data. The total (including all imaging modalities) imaging rate was lower in both groups at three months (11% in the intervention and 17% in the control group) than we expected in sample-size calculations (30%). This reduced the statistical power to detect significant differences at that time point. The results at three months indicated a trend towards less imaging in the intervention group. The effect sizes at 3 months and 12 months are very similar, despite differences in significance due to lower power at three months.

### Comparison with previous literature

To our knowledge, the effectiveness of an intervention developed to address both physician- and patient-related barriers to reducing the use of imaging has not previously been tested. Previous studies have demonstrated reduced imaging examinations with the inclusion of epidemiologic data in lumbar spine MRI reports [25–27]. In our study, information on the usefulness of imaging was provided to all the patients and professionals, regardless of whether or not imaging was undertaken.

Interesting findings from secondary outcomes include the reduction in sick leave days at 3- and 12-month follow-ups. According to Ree et al. (2016), a workplace educational back pain intervention also reduced sick leave for up to six months [28]. In addition, in an occupational setting, booklet information alone was cost-effective in comparison to no intervention among patients with mild LBP [29]. Decreasing imaging may also enhance reductions in sick leaves. Previous research showed that decreased imaging for LBP was associated with decreased health care utilization and sick leave days [30, 31]. In contrast to imaging, where an effect was observed only when a physician was the first contact, sick leave days also decreased when a physiotherapist was the first contacted professional. This is likely explained by physiotherapists’ ability to grant short-term (up to five days) sick leave due to LBP in Finland.

We used an average proportion of imaging (30%) for sample size calculation, which was later seen to be significantly higher than the proportion of imaging in the control group at 3 months (16.7%). According to previous literature, there is a wide variance in imaging proportions between different studies [5]. In this study, imaging proportions were likely decreased by the inclusion of physiotherapists, who can’t directly refer patients for imaging, as first contact health care providers. A larger portion of control group patients saw a physiotherapist first compared to the intervention group. When the first contacting professional was a physician, the proportion of imaging at 3 months was 29.4% (Table 5).

### Interpretation of findings

These results are consistent with the findings of the preliminary evaluation: the intervention reminded the professionals of the imaging guidelines and made it easier to follow them, and patient-related barriers were addressed by helping patients understand and receive an explanation for their pain [15]. It is possible that the shared-decision making and the written information supported decisions related to imaging use, and sustained, or even strengthened, the results over the 12-month follow-up. Common physician-reported reasons for using imaging include feeling that there is no alternative to imaging to offer the patient, and the lack of time to have a conversation with patients about diagnosis and why a scan is not needed [12]. The booklet provides practitioners with an alternative way to discuss the use of imaging with patients. It was easy to carry out, made sense to both the professionals and the patients, and supported evidence-based care.

The use of the patient education booklet in addition to usual care had no apparent effect on patients’ pain, physical functioning, disability or quality of life in comparison to usual care. This may have needed a more intensive and probably a more individualized intervention.

### Implications of the findings

The booklet appeared effective in reducing imaging and sick leave days among LBP patients in primary care and is suitable for implementation in similar primary care settings for this purpose. The patient education booklet is easy to use, inexpensive and seems to have no negative side effects, which endorses its implementation in practice. More research is needed to evaluate its effectiveness in different health care settings.

### Strengths and limitations

One strength of this study is that we used a cluster randomized design, and assessed the effectiveness of the booklet in an everyday clinical environment in the Finnish health care system. Several elements enhanced the generalization of the results and the possibility to use the intervention in different primary care organizations in future studies: the low number of exclusion criteria for LBP patients; inviting all physicians and physiotherapists in the study health care regions or organizations to participate; easy implementation procedure; low costs of the intervention; and minimal or no suspected harm related to the intervention.

A limitation of the current study was that some patients saw a physician first and others saw a physiotherapist first. We therefore conducted sensitivity analyses, which found a stronger effect on imaging rates when the booklet was used by a physician. In this subgroup, it additionally reduced subsequent LBP-related physician appointments in primary care by one third and LBP-related secondary health care appointments by about 10%. A systematic review and meta-analysis has earlier shown education for patients with acute or subacute LBP to reduce health care use (NNT 17 to prevent one primary health care physician visit due to LBP) [32].

The low number of clusters (eight) is also a limitation of the study. The health care policies of the clusters might have differed, even though we used suitable statistical methods for cluster randomized studies. For example, physicians’ sick-leave practices have shown to vary significantly [33]. In addition, no information was recorded on the number of eligible LBP patients who declined to participate in the study, or on whether the professionals neglected to invite some eligible patients to the study, despite being asked to recruit consecutive eligible patients. Both of these could have introduced selection bias. The loss to follow-up for the patient-reported outcomes is a major limitation in estimating PROMIS PF-20 change between baseline and three-month follow-up, one of the two primary outcomes, due to its potential to bias the results of this study. Therefore, conclusions related to the effect of the booklet on physical function are limited. Data collection from the electronic patient records in addition to the patient reported data is a strength of the study. Loss to follow-up for the other primary outcome, imaging at three months, was relatively low. The results and conclusions related to imaging, were not prone to the limitations potentially caused by loss to follow-up for the patient-reported outcomes.

The intervention group was provided with guideline recommendations as part of the training they received to use the booklet. Provision of guideline recommendations as a single strategy to reduce imaging for low back pain has not previously shown evidence of effectiveness [34], potentially because provision of guidelines does not sufficiently address all identified barriers to adherence to clinical guidelines [35]. The intervention in this trial was designed to address identified barriers, including both poor practitioner knowledge of guideline recommendations and pressure from patients to receive imaging. In the preliminary evaluation study 81% of professionals agreed that the booklet helped them to adhere to imaging guidelines [15]. The aim of this study was to assess the combination of the booklet and training session as a complete intervention, rather than identify the effect of the separate components.

One further limitation is that we did not evaluate how the health professionals used the booklet in the consultations or whether using the intervention increased consultation times, this would be an important area of future study.

## Conclusions

The booklet appeared effective in reducing the proportion of LBP patients who undergo imaging examinations when delivered by a physician. The intervention appeared to have no effect on PROMIS PF20 T-score change. The number of sick leave days appeared substantially lower in the intervention group.

## Supplementary Information

**Additional file 1.**

**Additional file 2.**

**Additional file 3.**

**Additional file 4.**

## Data Availability

The datasets used and analysed during the current study available from the corresponding author on reasonable request.

## Abbreviations

BBQ: Back Beliefs Questionnaire
CI: Confidence interval
CS: Cluster size
MID: Minimal Important Difference
CT: Computed tomography
CV: Coefficient of variation of cluster size
DE: Design Effect
DEPS: Depression Scale
EQ-5D: EuroQol EQ-5D-3L instrument
FABQ: Fear avoidance beliefs questionnaire
GEE: Generalized estimating equations
ICC: Intracluster Correlation Coefficient
ICERs: Incremental cost-effectiveness ratios
LBP: Low back pain
MID: Minimal Important Difference
MRI: Magnetic resonance imaging
NRS: Numeral Rating Scale
ODI: Oswestry Disability Index
ÖMPSQ: Örebro Musculoskeletal Pain Screening Questionnaire
OR: Odds ratio
PROMIS PF-20: Patient-Reported Outcomes Measurement Information System, 20-item physical functioning short form
PSEQ: Pain Self-efficacy Questionnaire
RMDQ-24: Roland Morris Disability Questionnaire 24 form
RR: Risk ratio
SBT: STarT Back Tool
SD: Standard deviation
