# Supplementary material for: Patient education booklet to support evidence-based low back pain care in primary care – a cluster randomized controlled trial
Source: BMC Fam Pract. 2021 Sep 7;22:178. doi: 10.1186/s12875-021-01529-2 (PMC8422671; doi:10.1186/s12875-021-01529-2)
Supplement: Supplementary file 1 — Additional file 1. [file 12875_2021_1529_MOESM1_ESM.pdf]

Patient education booklet to support evidence-based low back pain care in primary care – a cluster randomized controlled trial

Anna Sofia Simula, University of Oulu and ESSOTE

Globally, low back pain is the heaviest burden for health care and society

Maher et al. Non-specific low back pain: Lancet 10/2016

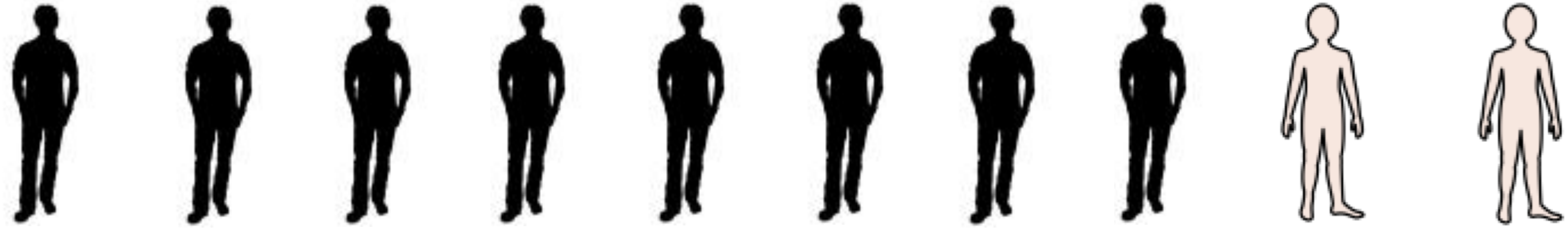

Lifetime prevalence

Benefits paid by Kela in 2016; 1 000€

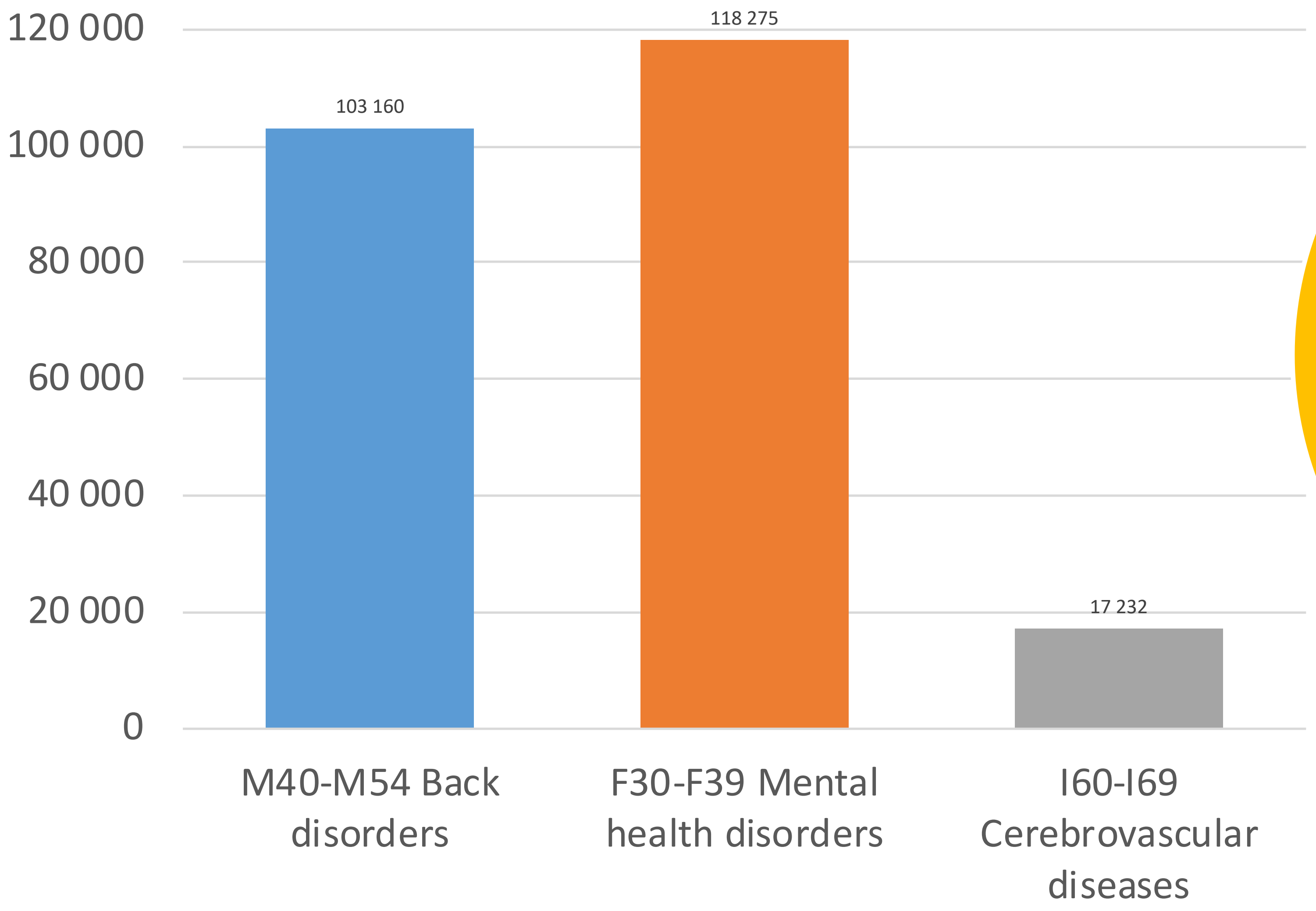

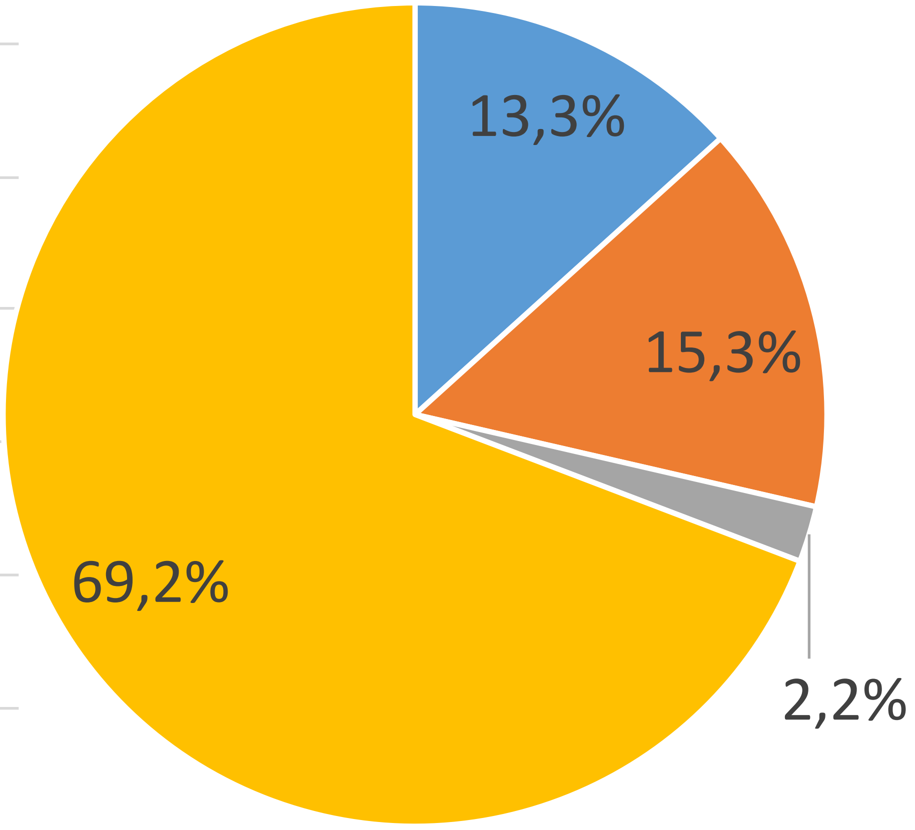

Kela sickness insurance statistics (20.9.2017)

Physician appointments ICD10: M40-M54
ESSOTE 2015/EKSOTE 2016

(Occupational health care in ESSOTE health care region about 5000 LBP-related physician appointments per year)

	Population	18–65 years %
ESSOTE	79 808	57.2
EKSOTE	131 764	58.6

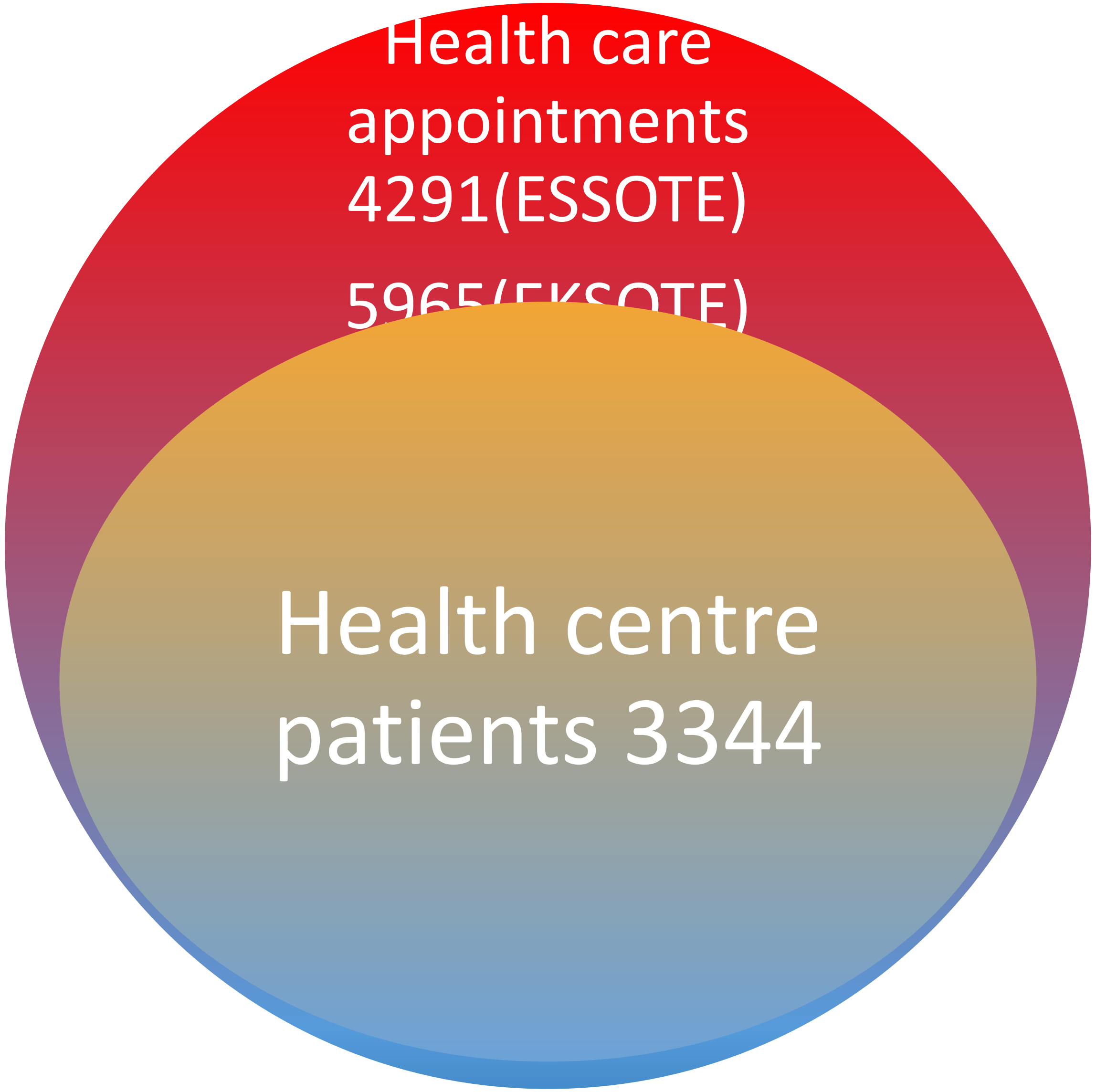

Over 90% of LBP patients have non-specific LBP

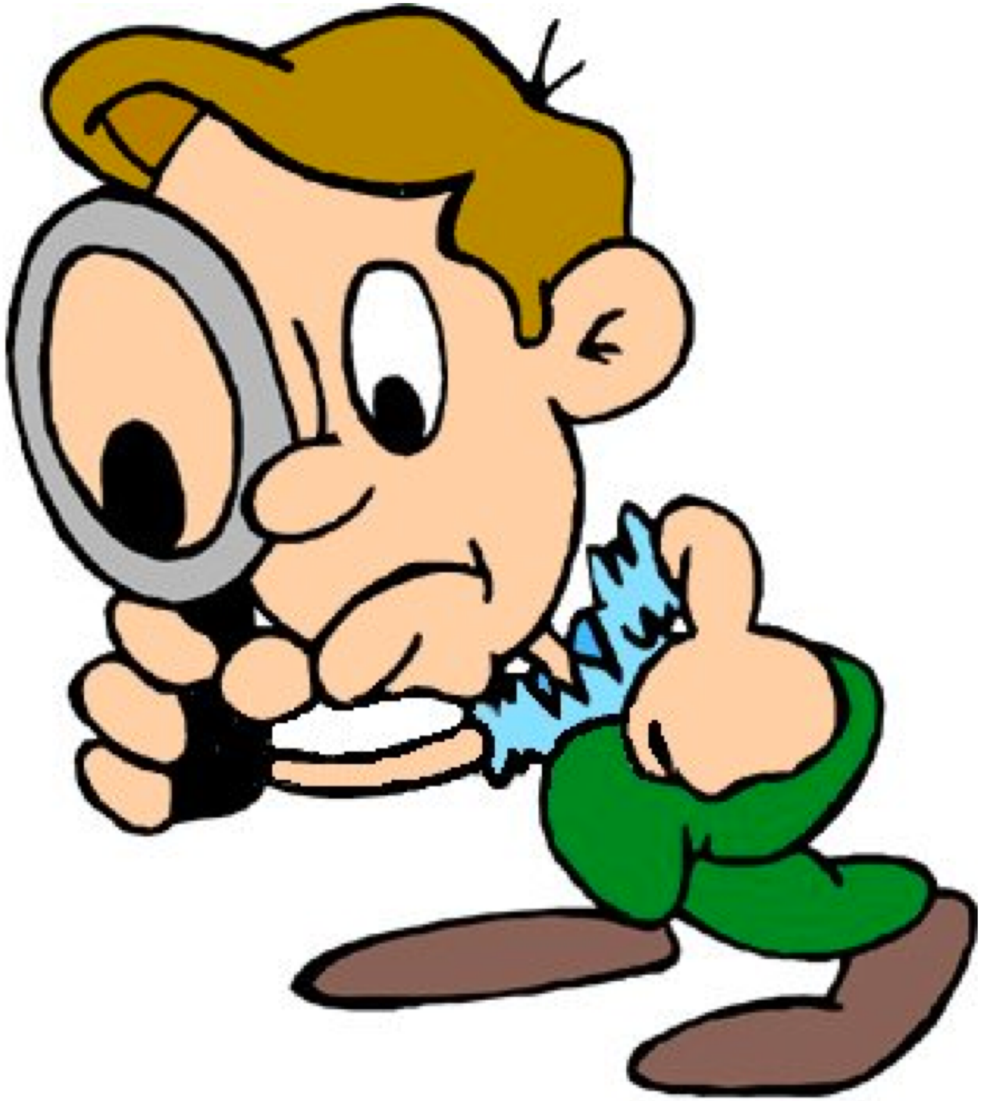

Hartvigsen J et al. on behalf of the Lancet Low Back Pain Series Working Group. What low back pain is and why we need to pay attention. Lancet 2018; 391(10137):2356-67.

As a symptom, LBP is a complex biopsychosocial entity

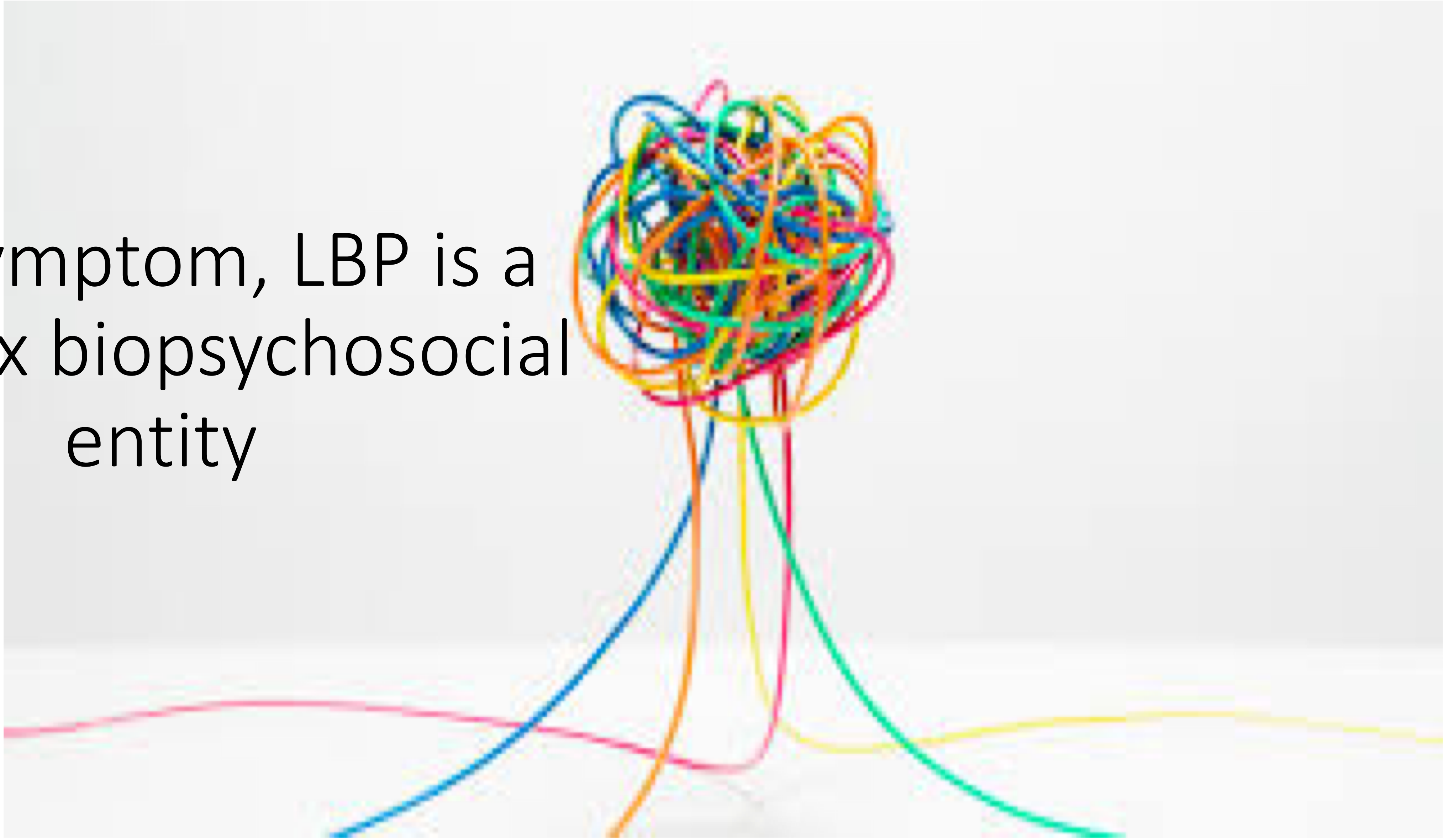

Strong risk factors for prolonged pain

- pain intensity
- belief related to prolonged pain
- low self-efficacy
- passive coping strategies
- high level of catastrophizing
- fear-avoidance beliefs
- depression
- sleep problems
- stress
- low education
- low social class
- unemployment

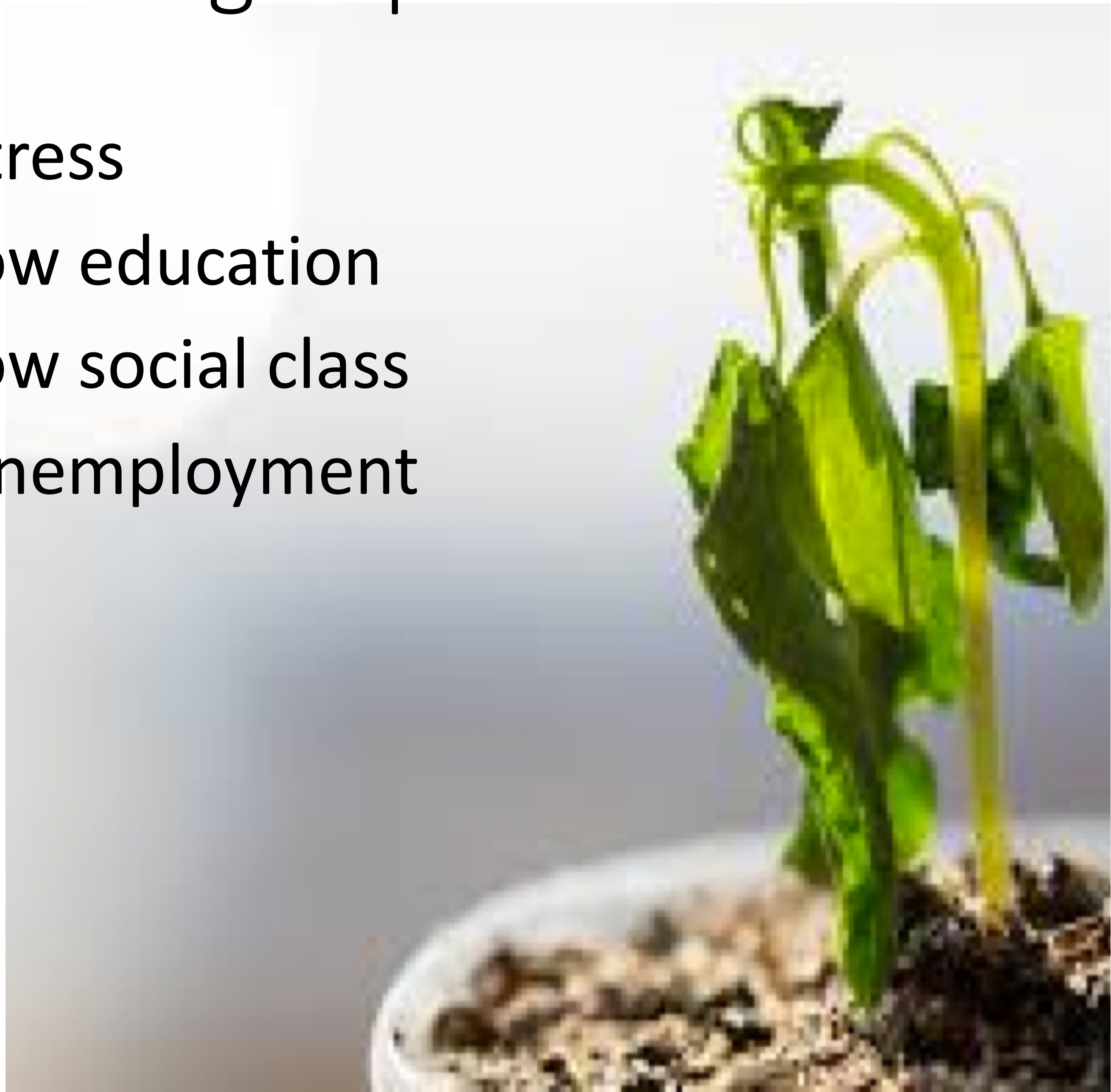

What kind of care and for whom?

- therapeutic exercises
- physiotherapy
- manual therapy
- patient education
- cognitive behavioral therapy
- acupuncture
- pain medication

No-one is better than anyone else at the group level

Suomalaisen Lääkäriseura Duodecimin ja Suomen Fysiatriayhdistyksen asettama työryhmä. Alaselkäkipu. Käypä hoito -suositus 27.4.2015.

O'Keeffe et al.: Comparative Effectiveness of conservative interventions for nonspecific chronic spinal pain: physical, behavioral/ psychologically informed, or combined? A systematic review and meta-analysis J Pain 2016; 17:755-74

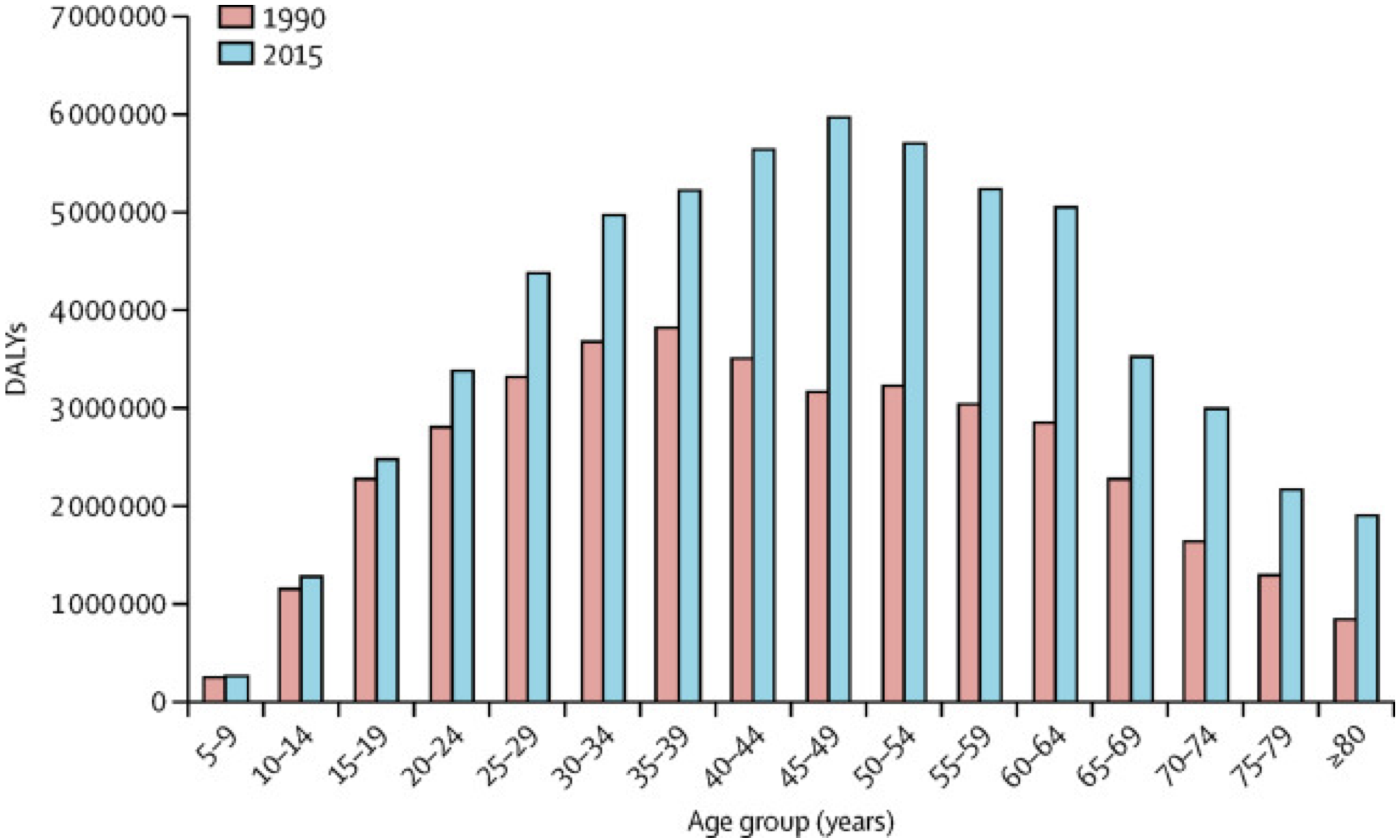

Hartvigsen J et al. on behalf of the Lancet Low Back Pain Series Working Group. What low back pain is and why we need to pay attention. Lancet 2018; 391(10137):2356-67.

Something must be done!

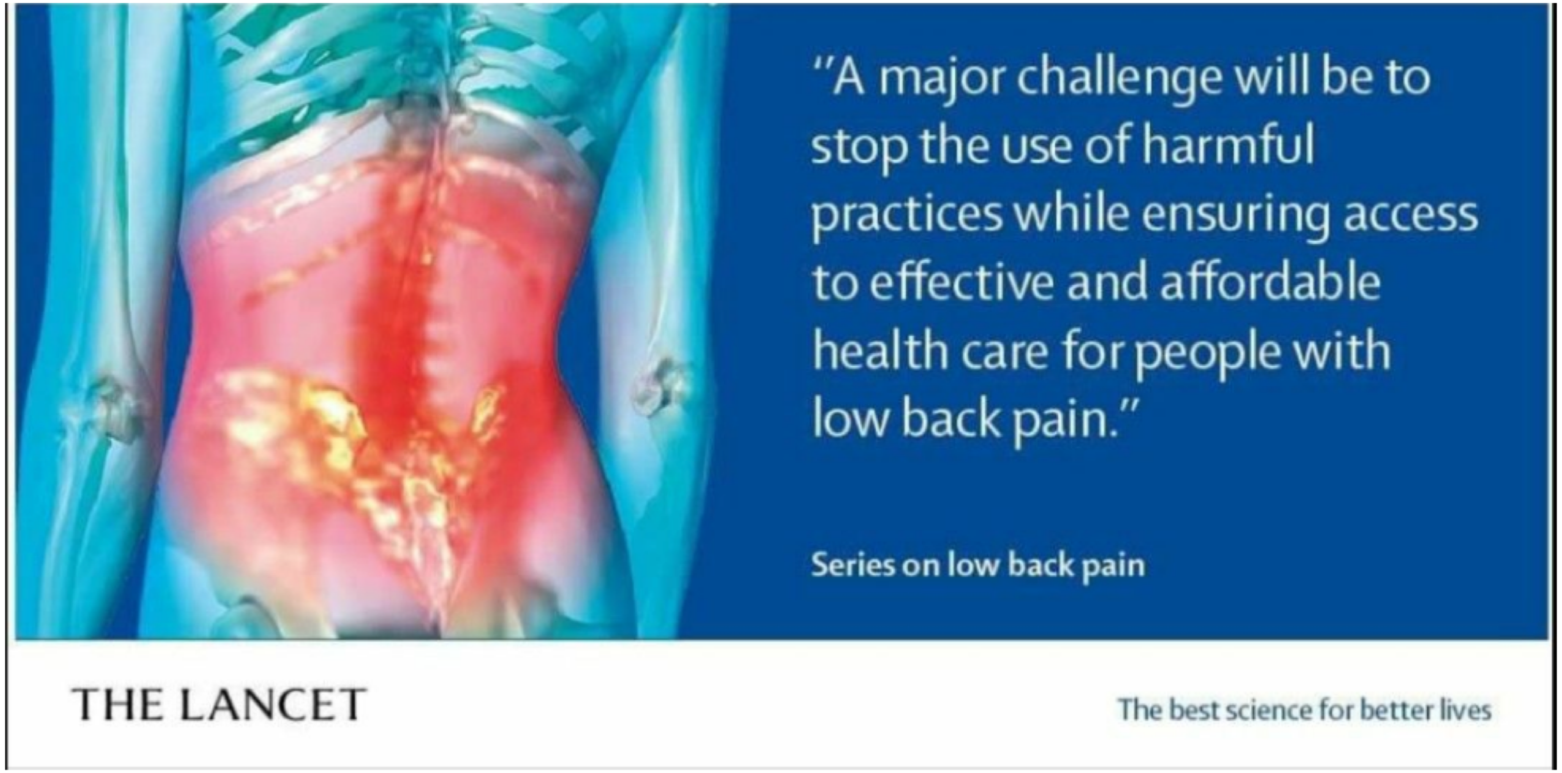

Patient education booklet as part of care

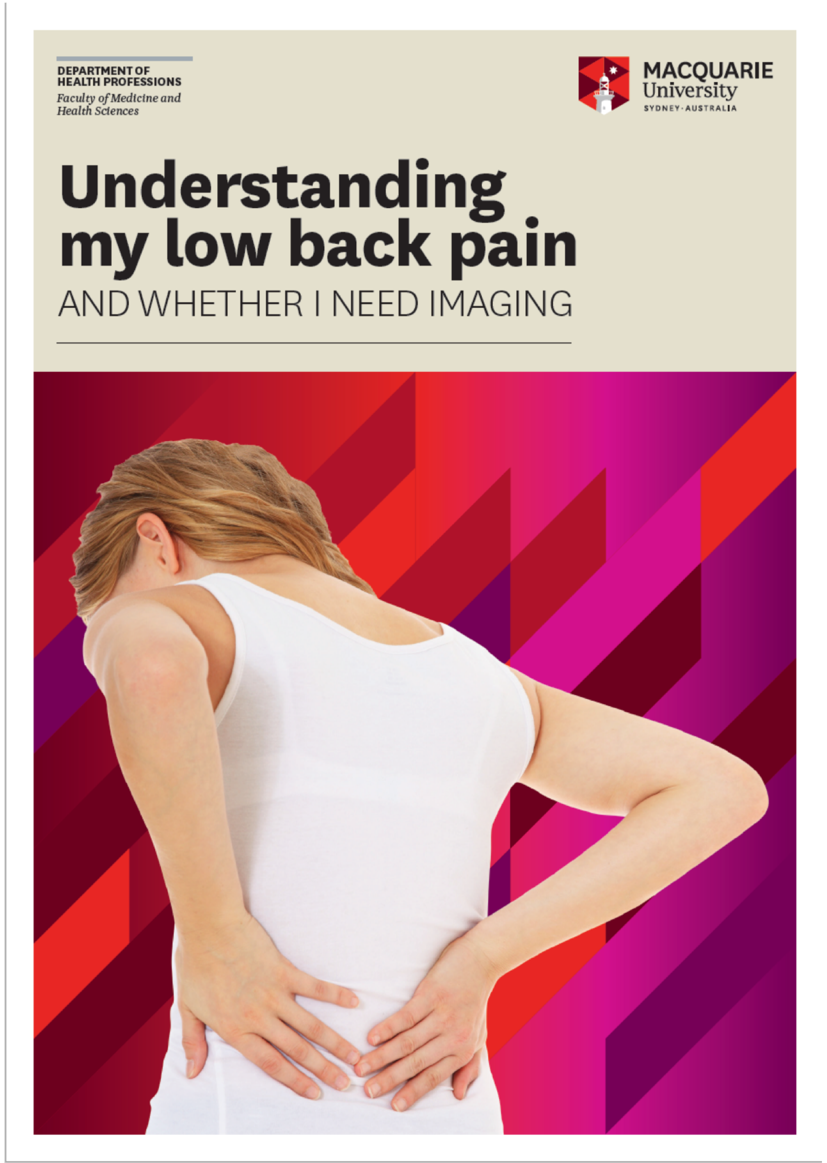

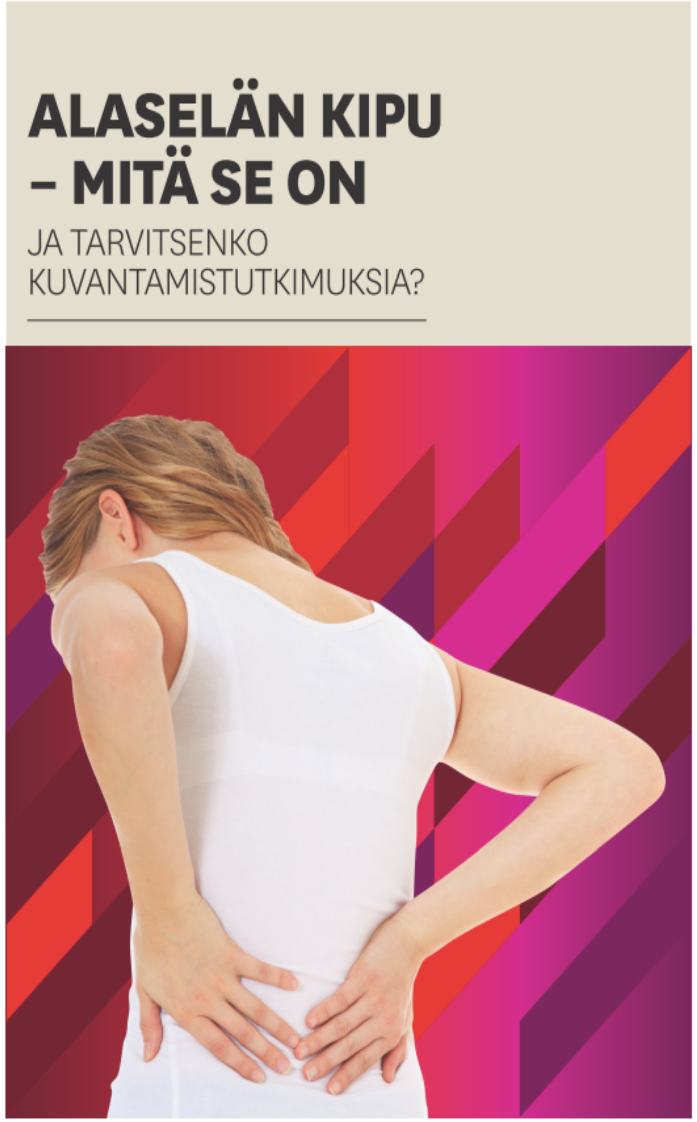

Why a booklet?

- Previous study found that a booklet was cost-effective in mild LBP patient cases (Rantonen et al._ BCM BublicHealth 2016_Cost-effectiveness)
- Supports consistent communication
- Easy and inexpensive intervention

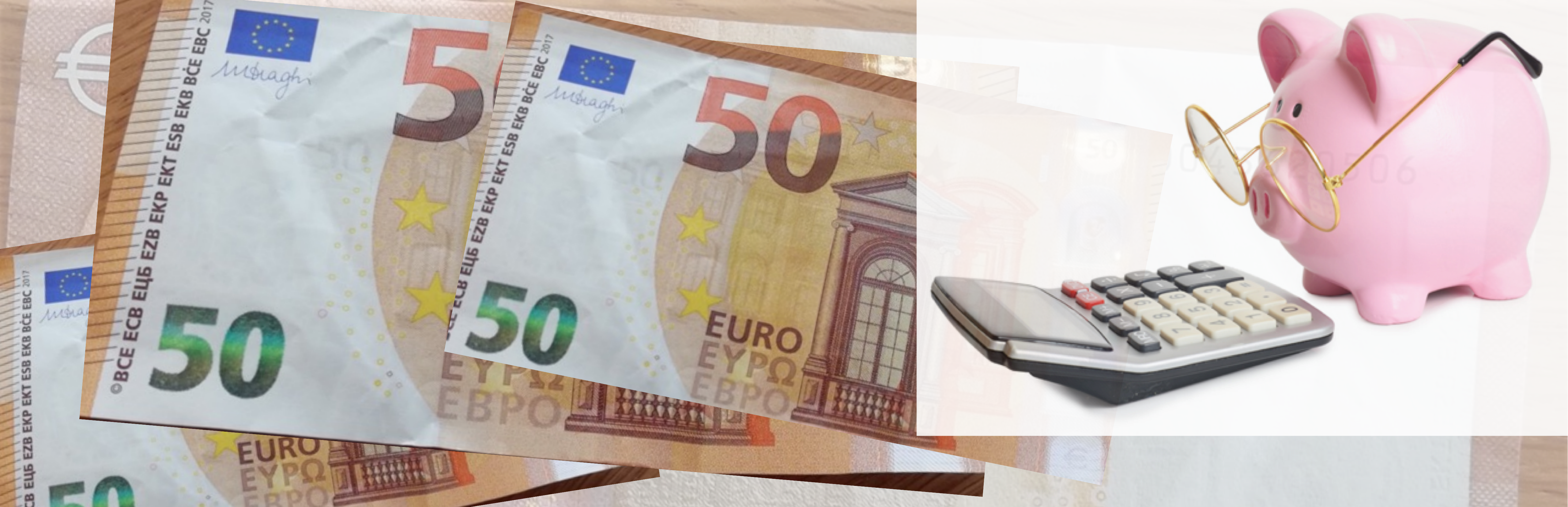

# Compared to NC, the Booklet reduced HC costs by 196€ and SA by 3.5 days per year.

Rantonen et al._ BCM BublicHealth 2016_Cost-effectiveness of providing patients with information on managing mild lowback symptoms in an occupational health setting.

Face-to-face patient information given by an OH nurse in addition to a booklet was no more effective than the booklet alone in treating employees with mild LBP in an OH setting.

Rantonen et al._Scand J Work Environ Health 2014;40(2)-156-166 Face-to-face information combined with a booklet versus a booklet alone for treatment of mild low-back pain- a randomized controlled trial

Review Article

Barriers to Primary Care Clinician Adherence to Clinical Guidelines for the Management of Low Back Pain

A Systematic Review and Metasynthesis of Qualitative Studies

Susan C. Slade, PhD,† Peter Kent, PhD,‡ Shilpa Patel, DHealth Psy,§*
Tracey Bucknall, PhD,‖¶ and Rachelle Buchbinder, PhD†*

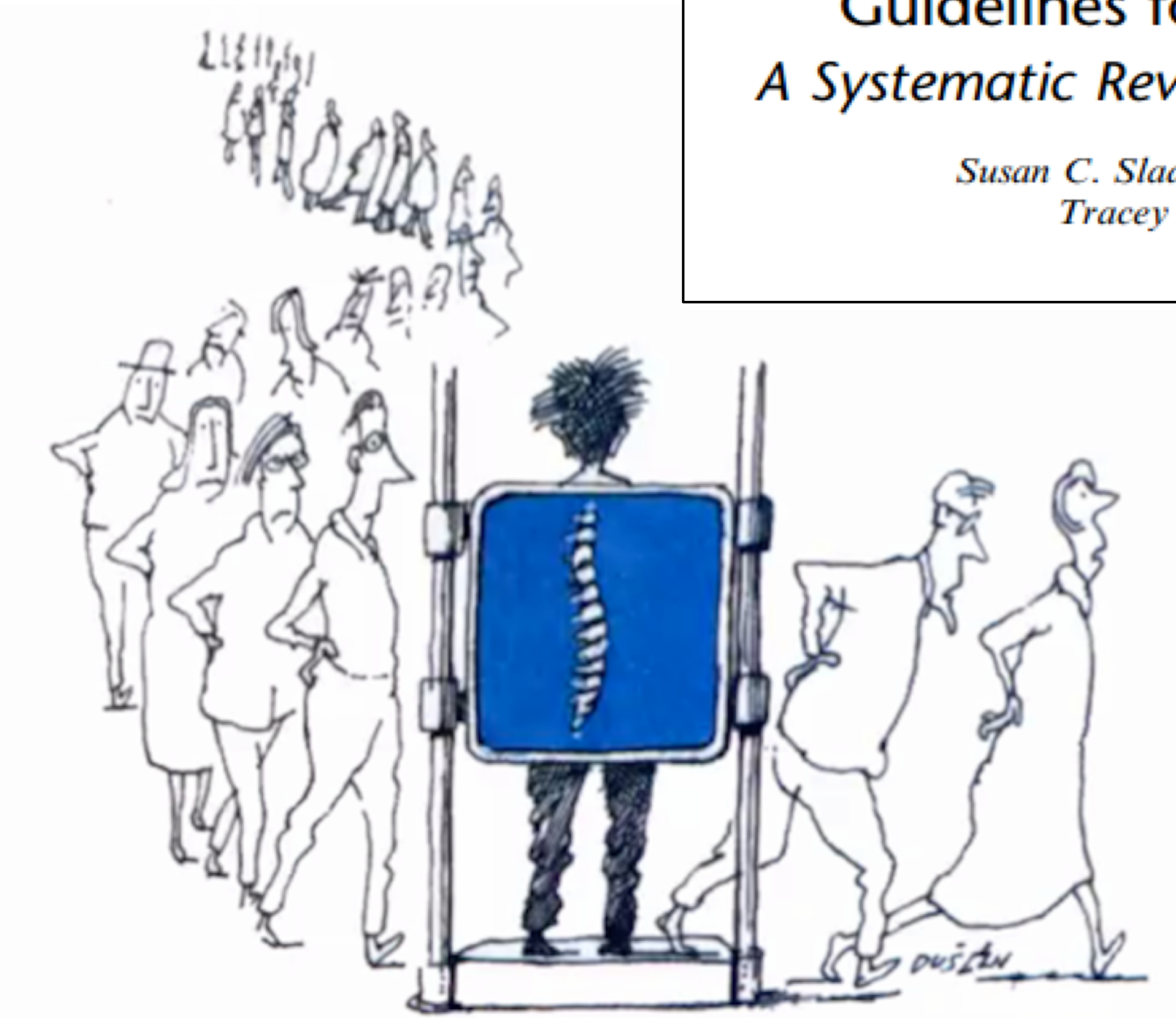

Transcultural adaptation and validation of Booklet

- Original version developed in Macquarie University, Sydney, Australia (by Prof. **Mark Hancock** and **Hazel Jenkins**)
- Questionnaire as preliminary test
- 136 patients
- 32 physicians and physiotherapists

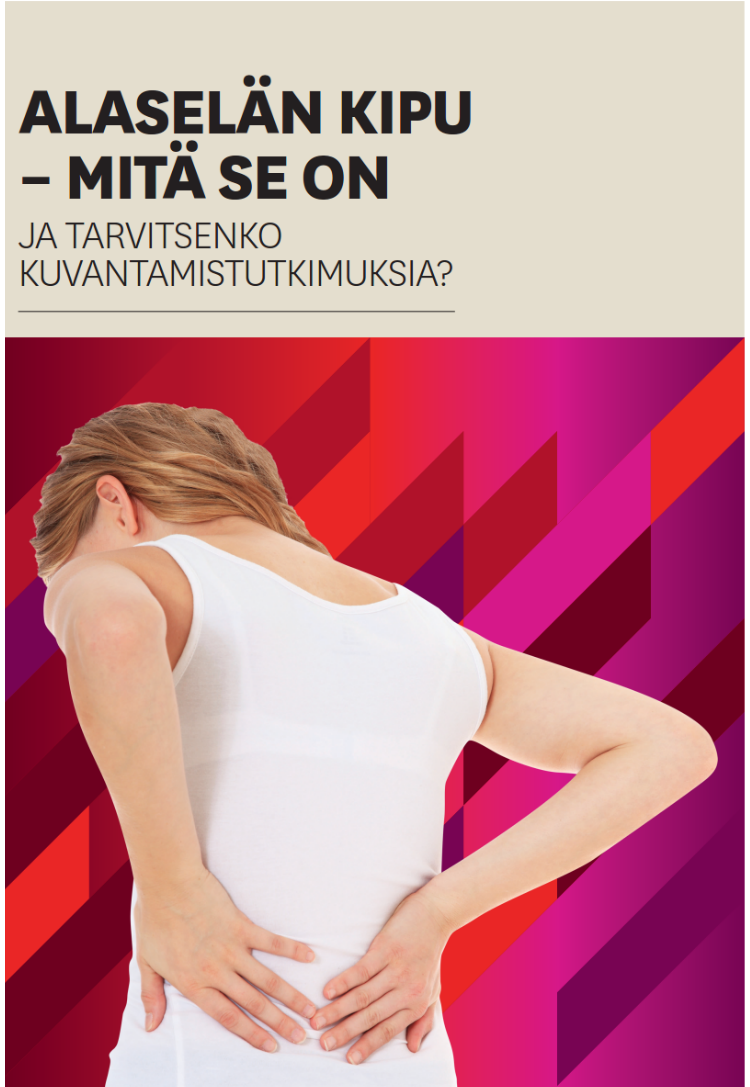

Patient evaluation of benefits of Booklet on basis of statements, % of responses (n=136)

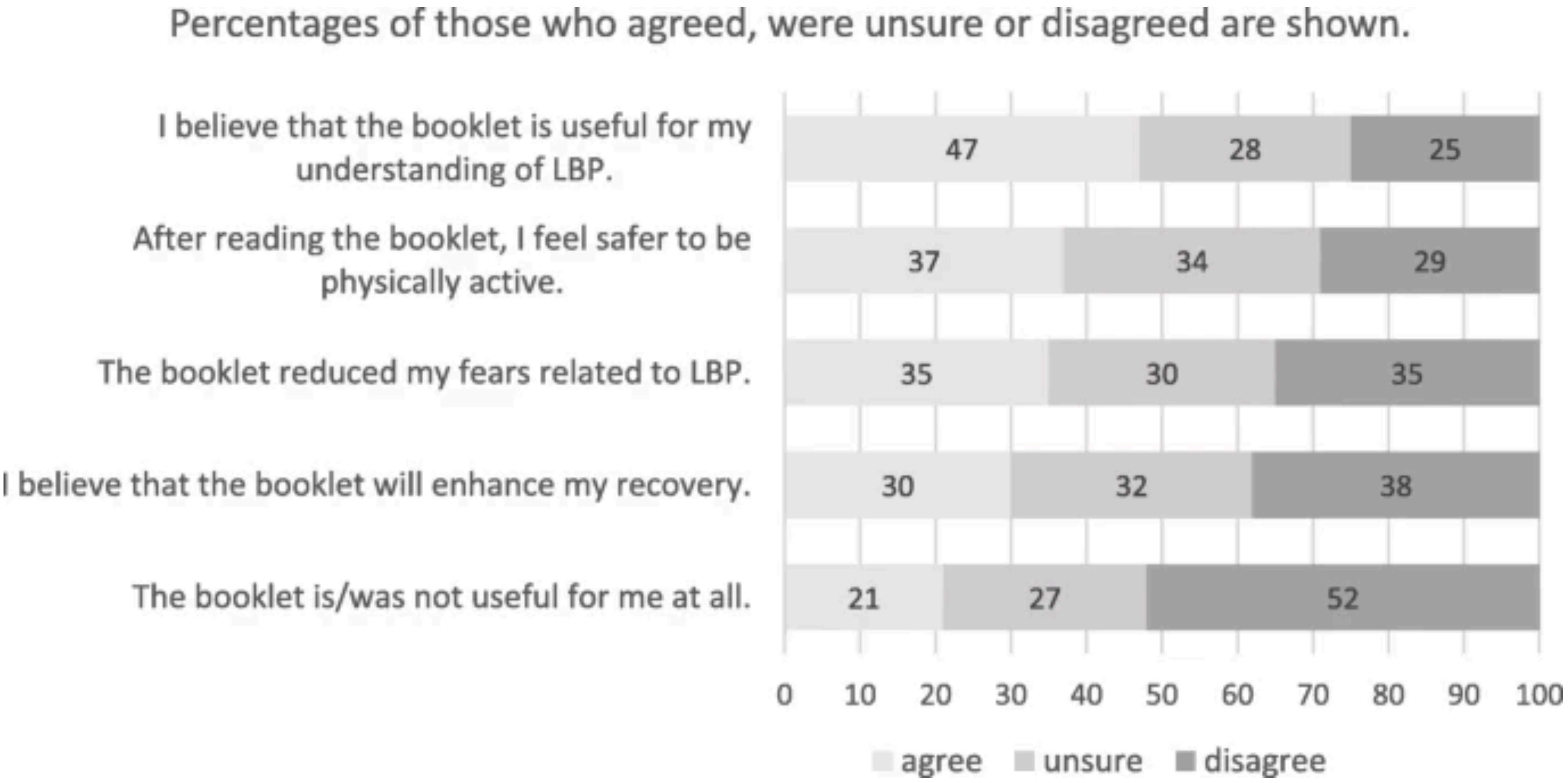

Cluster randomized study of Booklet's effectiveness

ESSOTE
EKSOTE
Rovaniemi
Hyvinkää
Kerava
ESTT
TT-Mikkeli

Clusters (n=8)

Pair-wise randomization

Intervention

(use of booklet + 30min training for professionals)

3 primary health care regions
1 occupational health servoce organization

Control

Usual care without booklet

4 primary health care regions
1 occupational health service organization

Study population

Inclusion criteria

- Low back pain
- Age 18–65 years

Exclusion criteria

- Age under 18 or over 65 years
- First time patient presents to health care for LBP lasting 2 weeks or less
- Suspected serious cause of LBP (for example, cancer, fracture or infection), LBP requiring urgent (emergency) care, such as cauda equina syndrome)

What does participation in the study mean for the patient?

- They receive a link to the questionnaire (mostly multiple choice questions) via emaoö
  - baseline
  - 3 months
  - 1 year
- Participation will not affect their care
- They will not receive compensation
- They will be involved in improving health care system for LBP patients

What is measured?

Patients

- Affect on fuctional capacity (PROMIS + ODI)
- Quality of life (EQ-5D)
- Use of health care services
- Sick leaves

Professionals

- back pain beliefs and attitudes
- Feasibility of model

Costs

- Use of health care services
- imaging examinations
- pain medication
- sick leaves

Recruitment

1. Explain that research on the development of LBP treatment is underway, and that the study does not affect the patient's treatment. Follow-up involves electronic questionnaires.
2. Provide a patient information leaflet and consent form
3. Return signed consent forms to the study group

https://drive.google.com/open?id=1DQ9r9JKr7M-PG2uKL5jZgEwfl0bI-sjw

(Link to materials: patient information and consent forms, video of example recruitement, inclusion and exclusion criteria, guide on how to return signed consent forms)

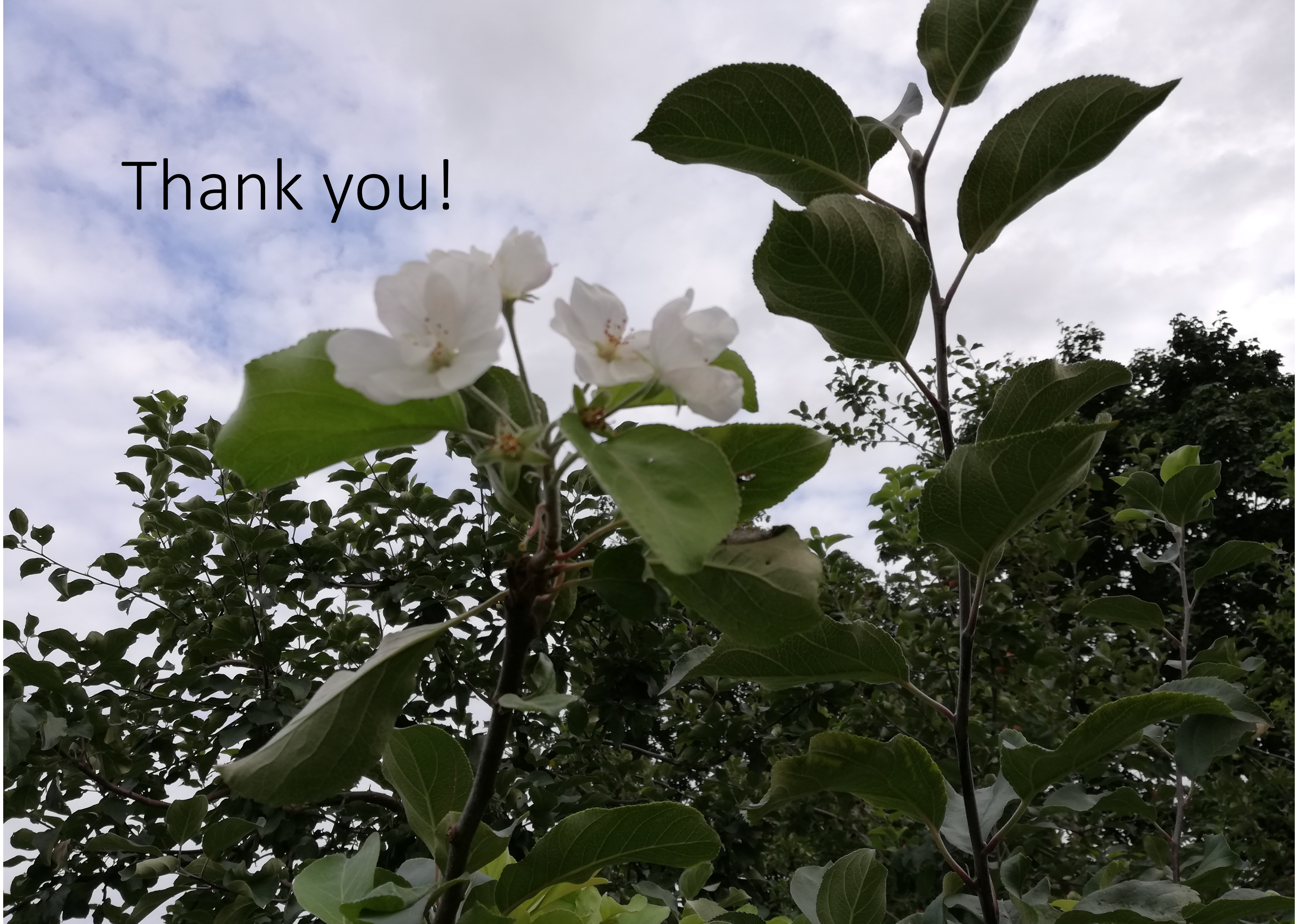
Thank you!
