# Supplementary material for: Patient education booklet to support evidence-based low back pain care in primary care – a cluster randomized controlled trial
Source: BMC Fam Pract. 2021 Sep 7;22:178. doi: 10.1186/s12875-021-01529-2 (PMC8422671; doi:10.1186/s12875-021-01529-2)
Supplement: Supplementary file 2 — Additional file 2. [file 12875_2021_1529_MOESM2_ESM.pdf]

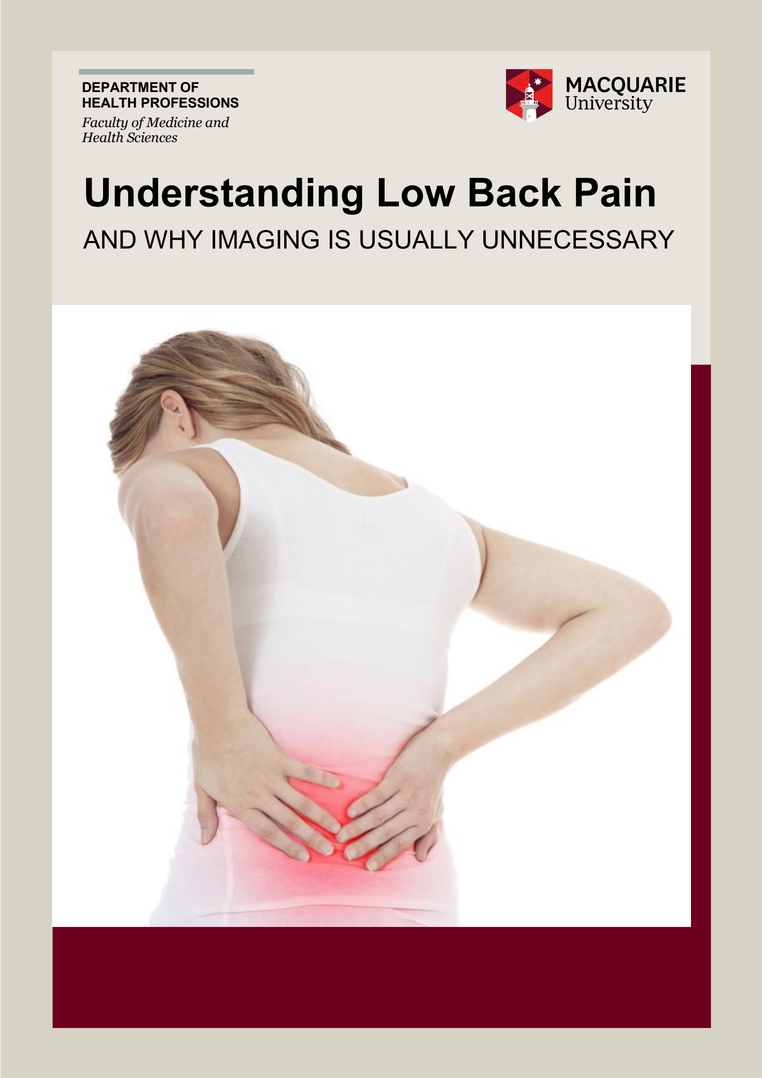

Patient education booklet to support evidence-based low back pain care in primary care – a cluster randomized controlled trial

Anna Sofia Simula

Why a booklet?

- Previous study found that a booklet was cost-effective in mild LBP patient cases (Rantonen et al._ BCM BublicHealth 2016_Cost-effectiveness)
- Supports consistent communication
- Easy and inexpensive intervention

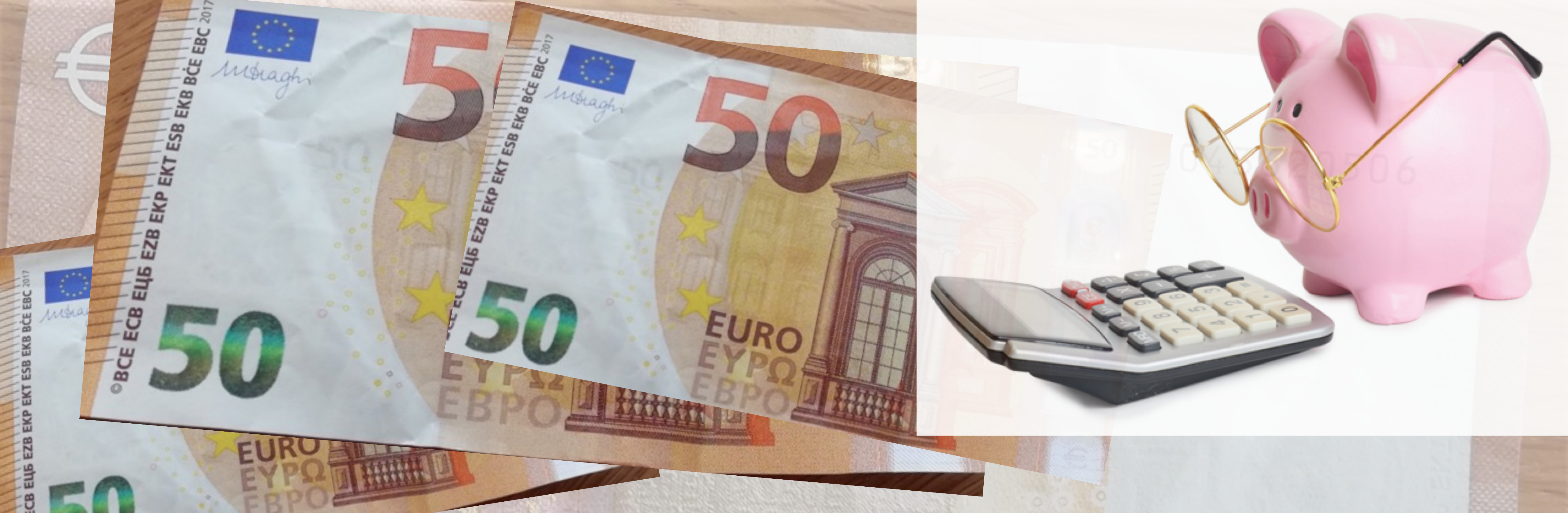

# Compared to UC, the Booklet reduced HC costs by 196€ and SA by 3.5 days per year.

Rantonen et al._ BCM BublicHealth 2016_Cost-effectiveness of providing patients with information on managing mild lowback symptoms in an occupational health setting.

Face-to-face patient information by an OH nurse in addition to a booklet was no more effective than the booklet alone in treating employees with mild LBP in an OH setting.

Rantonen et al._Scand J Work Environ Health 2014;40(2)-156-166 Face-to-face information combined with a booklet versus a booklet alone for treatment of mild low-back pain- a randomized controlled trial

Barriers and facilitators to implementing a booklet aiming to reduce imaging in primary care: a qualitative study with providers and patients

Hazel Jenkins, Mark Hancock, Niamh Moloney, Simon French, Chris Maher, Blake Dear, Simon Willcock, John Magnussen

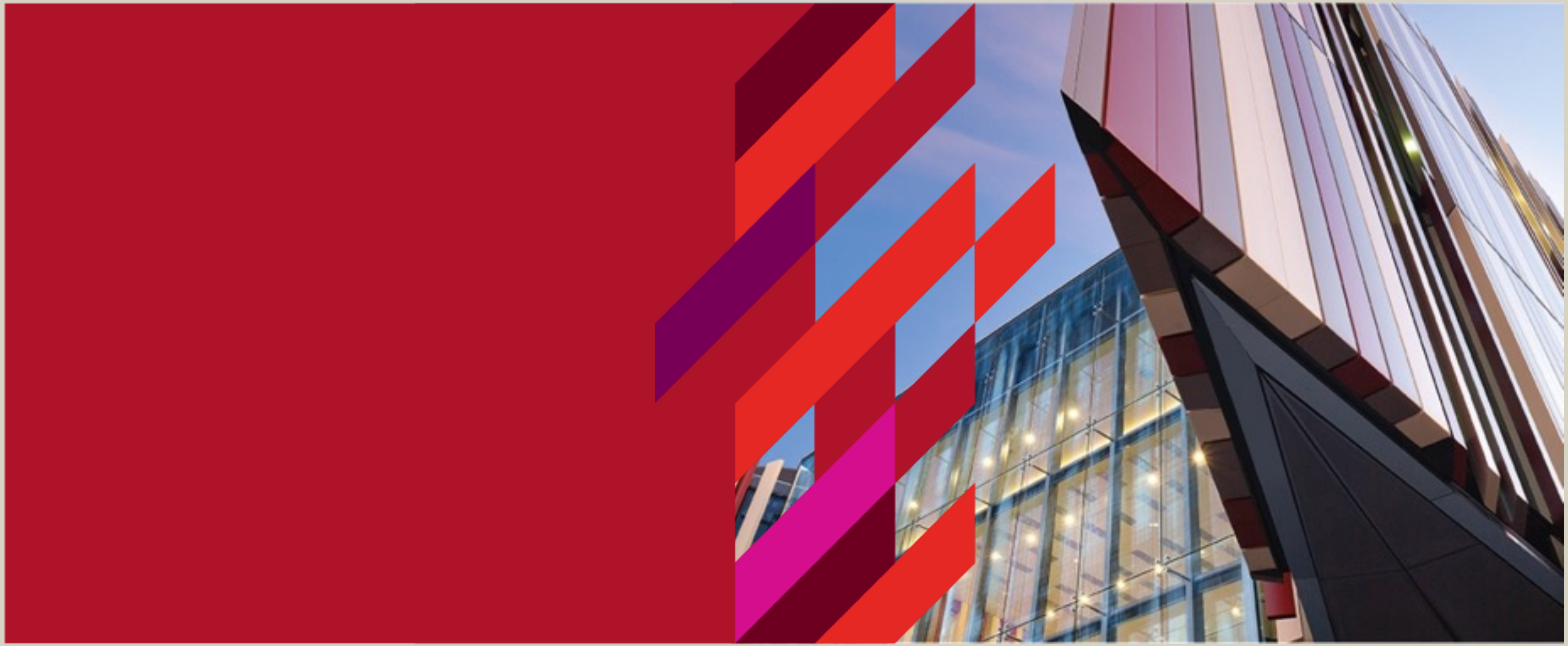

CMAJ RESEARCH

Effectiveness of interventions designed to reduce the use of imaging for low-back pain: a systematic review

Hazel J. Jenkins MChir, Mark J. Hancock PhD, Simon D. French PhD, Chris G. Maher PhD, Roger M. Engel PhD, John S. Magnussen PhD

Background

REVIEW ARTICLE

Barriers to Primary Care Clinician Adherence to Clinical Guidelines for the Management of Low Back Pain

A Systematic Review and Metasynthesis of Qualitative Studies

Susan C. Slade, PhD,† Peter Kent, PhD,‡ Shilpa Patel, DHealth Psy,§
Tracey Bucknall, PhD,‖¶ and Rachelle Buchbinder, PhD*†*

- Clinicians use imaging referrals to manage clinical consultation

Do you have symptoms indicating a possible serious cause for your low back pain? (eg. cancer, infection or fracture)

Yes → Imaging or further investigation may be needed, if recommended by your GP

No ↓

Is there possible irritation or compression to the nerves to the legs?

Yes → Imaging or further investigation are only needed if surgery is being considered

No ↓

Simple low back pain: Imaging or further investigation are not required

→ If symptoms get worse, or are not improving, within 2 weeks – see your GP to discuss your plan

All about low back pain

WHAT IS CAUSING MY LOW BACK PAIN?

Common causes of low back pain include minor soft tissue injuries such as muscle spasm or joint sprain.

Less than 1 in 100 people with low back pain have a serious cause for their pain (eg, fracture, cancer, infection or inflammatory arthritis).

These conditions are usually obvious and your GP will take a clinical history and perform an assessment to 'check' for them.

I HAVE STRONG PAIN – DOESN'T THIS MEAN A BAD INJURY HAS OCCURRED?

No – lots of research shows that strong pain does not equal a bad injury.

Many people experience a lot of pain. This does not mean there is any damage in their back.

Some factors that can change the pain you feel include your mood and stress levels, your sleep quality and your lifestyle and general fitness levels.

HOW COMMON IS SIMPLE LOW BACK PAIN?

It is common and often reoccurs.

Four out of five people will get low back pain at some time in their life. Half of these will go on to get further episodes of back pain throughout their life.

Recurrence is common and does not mean that there is greater reason for concern.

WHAT IS SIMPLE LOW BACK PAIN?

Low back pain, where **no indication of a serious cause can be found.**

Pain may be mild or very strong, but it **typically improves a lot over the first couple of weeks.**

There may also be pain into the legs. **As the back gets better, the leg pain usually improves too.**

Why isn't imaging needed?

Imaging (eg, x-rays, CTs or MRI scans) doesn't usually **help find the cause of pain.**

The **treatment and speed of recovery** for most cases of back pain **is the same whether imaging is used or not.**

WON'T IMAGING SHOW WHAT IS CAUSING MY LOW BACK PAIN?

No - the underlying cause of back pain **cannot** usually be seen on imaging.

Imaging can help diagnose serious causes of low back pain - but, these are very rare and your GP will check for signs of them.

I KNOW OTHER PEOPLE WHO FOUND 'CHANGES' ON IMAGING OF THEIR BACK – WHAT IF I HAVE THESE 'CHANGES' TOO?

Many 'changes' can be seen on imaging, but it is unknown if they are causing your pain.

Most of the 'changes' seen on imaging are normal and more common the older you get.

Even people **without back pain** commonly have imaging 'changes'. For example, 6 out of 10 middle aged people without back pain have changes on imaging such as disc bulges or degeneration.

WHY SHOULDN'T I GET IMAGING 'JUST IN CASE'?

Unnecessary imaging has some **risks:**

- **Radiation** exposure (for x-ray and CT) can **increase the risk of cancer.** Less radiation is better
- It can **cost you money** and is **time consuming**
- **Changes on imaging are often seen and may cause stress, anxiety and worry,** even though they are usually unimportant
- Imaging has been associated with **worse patient outcomes** and an increase in **unnecessary surgery**

MRI findings among patients without LBP

	20-year-olds	40-year-olds	80-year-olds
Disk degeneration	37%	68%	96%
Disk protrusion	30%	50%	84%
Annular fissure	19%	22%	83%
Facet degeneration	4%	18%	83%
Spondylolisthesis	3%	8%	50%

AJNR Am J Neuroradiol. 2015 Apr;36(4):811-6. doi: 10.3174/ajnr.A4173. Epub 2014 Nov 27.
Systematic literature review of imaging features of spinal degeneration in asymptomatic populations. Brinjikji W et al.

What can I do to help decrease my low back pain?

The best thing you can do is to **stay as active** as possible.

HOW WILL ACTIVITY HELP MY LOW BACK PAIN?

Activity keeps the back mobile, flexible and strong. **Movement, even if it causes temporary mild pain, is good for your back.**

Research shows that bed rest or prolonged inactivity can delay recovery.

WHAT EXERCISES CAN I DO WHEN I HAVE LOW BACK PAIN?

Any exercise you enjoy can help decrease your pain and get you moving.

Some suggestions include:

- **Gentle stretches or exercises** (such as Tai Chi or Yoga)
- Aerobic exercises like **walking or swimming**

You can also ask your GP for some safe exercises and stretches if you are unsure.

IS THERE ANYTHING ELSE I CAN DO?

- Try to stay positive and take steps towards a healthier lifestyle.
- When you feel stressed, angry or worried your pain might feel worse. Managing these feelings might help your low back pain.
- Lifestyle factors may also be associated with low back pain. You might find it helpful to address poor sleep patterns and consider changes to diet, alcohol intake and smoking.

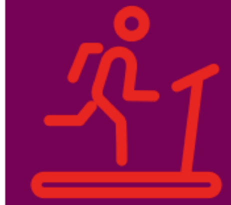

MY BACK HURTS – HOW CAN I STAY ACTIVE?

You may need to take things a bit easier for a few days, but **you also need to get back into your normal activities as soon as possible.**

Normal activities include your regular work, household and recreational activities.

Staying active is safe with simple low back pain – even if there is some pain.

What is my low back pain management plan?

THE FOLLOWING SUGGESTIONS MAY HELP REDUCE YOUR PAIN AND INCREASE YOUR RECOVERY:

- Take it easy for the first day or two after the pain starts if needed, then start getting back into your normal daily activities as soon as possible.
- Try to avoid staying in one position for a prolonged time.
- Try some gentle exercise such as walking, swimming or stretching.

POSSIBLE PAIN-RELIEF STRATEGIES RECOMMENDED BY YOUR GP:

- [ ] Pain-relief medications: ____________________
- [ ] Heat
- [ ] Referral to a physiotherapist / chiropractor / osteopath / other

OTHER SUGGESTIONS FROM YOUR GP (EG. SPECIFIC EXERCISES, STRESS RELIEF, SLEEP STRATEGIES ETC.)

What should I do next?

- ☐ Please come back in ______ for a review
- ☐ Please come back if your pain is not improving after ______ weeks
- ☐ As long as your pain is improving you do not need to return for a review

WHEN SHOULD I RETURN FOR FURTHER MEDICAL ADVICE?

Occasionally low back pain does not improve or symptoms may change.

CONTACT YOUR HEALTH CARE PRACTITIONER IF YOU HAVE ANY OF THE FOLLOWING SYMPTOMS:

1. Difficulty passing or controlling urine or stool
2. Numbness around your back passage or genitals or in both legs
3. Unsteadiness on your feet
4. Pain which gets worse rather than better over several weeks
5. Feeling generally unwell with your low back pain (for example: fever or unexplained weight loss)

Where can I find more information about low back pain?

There are a number of resources on the internet you can access to get quality information about the diagnosis and treatment of low back pain. Some suggested online resources include:

[illegible] Australia

MISTÄ VOIN LÖYTÄÄ LISÄTIETOA ALASELÄN KIVUSTA?

Internetissä on useita luotettavia sivustoja, joissa on tietoa selkäkivun diagnosoinnista ja hoidosta.

Käypä hoito -suositus Alaselkäkipu: **http://bit.ly/kaypahoitosuositus**

Terveyskirjasto: **http://bit.ly/terveyskirjasto**

Ota kipu haltuun: **http://www.otakipuhaltuun.fi/**

www.retrainpain.org

ACKNOWLEDGEMENTS

The following information sources were used in the development of this booklet:

- Burton K, Klaber Moffett J, Main C, Roland M, Waddell G, The Back Book 2nd Edition (2002), The Stationary Office, England
- National Health and Medical Research Council: Acute Low Back Pain
- Orthopaedic Spinal Services, SA Health: Advice for Managing Low Back Pain
- Orthopaedic Spinal Services, SA Health: Scans and Low Back Pain
- NPS MedicineWise: Back Pain Choices
