# Supplementary material for: Patient education booklet to support evidence-based low back pain care in primary care – a cluster randomized controlled trial
Source: BMC Fam Pract. 2021 Sep 7;22:178. doi: 10.1186/s12875-021-01529-2 (PMC8422671; doi:10.1186/s12875-021-01529-2)
Supplement: Supplementary file 3 — Additional file 3. [file 12875_2021_1529_MOESM3_ESM.docx]

**Additional file 3** Checklist for electronic patient record data.

| ID | | |
| --- | --- | --- |
| Date of consent | | |
| First contacted professional:  physician/nurse and physician/physiotherapist/nurse alone | | |
|  | | |
| **Number of LBP-related appointments** | **3 months** | **12 months** |
| Physiotherapist |  |  |
| Nurse |  |  |
| Physician |  |  |
| Physiatrist |  |  |
| Orthopaedist |  |  |
|  |  |  |
| All primary care contacts |  |  |
| Number of LBP-related sick leave days |  |  |
|  | | |
| RTG (X-ray) yes/no |  |  |
| MRI yes/no |  |  |
| CT yes/no |  |  |
