# Supplementary material for: Patient education booklet to support evidence-based low back pain care in primary care – a cluster randomized controlled trial
Source: BMC Fam Pract. 2021 Sep 7;22:178. doi: 10.1186/s12875-021-01529-2 (PMC8422671; doi:10.1186/s12875-021-01529-2)
Supplement: Supplementary file 4 — Additional file 4. [file 12875_2021_1529_MOESM4_ESM.docx]

**Additional file 4** Sensitivity analysis of effects of using patient education booklet during low back pain patient appointment when first contacted professionals were nurse and physician together. Limited number of participants – more research on this group is needed.

| **First contacted professional** | **Nurse and physician n=23/17*** | | | |
| --- | --- | --- | --- | --- |
| **Imaging (yes/no)^1^** | **Intervention %(n)** | **Control %(n)** | **OR (95% CI)** | **P value** |
| Imaging 3 months | 26.1 (6) | 17.6 (3) | **2.60 (1.87-3.62)** | **<0.001** |
| Imaging 12 months | 26.1 (6) | 43.8 (7) | **0.61(0.55-0.69)** | **<0.001** |
| *Radiographs 3 months* | 8.7 (2) | 0.0 (0) | ** |  |
| *Radiographs 12 months* | 8.7 (2) | 0 (0) | ** |  |
| *Magnetic resonance imaging* (*MRI) 3 months* | 17.4 (4) | 17.6 (3) | **1.54 (1.13-2.10)** | **0.006** |
| *MRI 12 months* | 17.4 (4) | 41.2 (7) | **0.40 (0.36-0.44)** | **<0.001** |
|  |  |  |  |  |
| **Sick leave days^2^** | **mean (SD)** | **mean (SD)** | **RR (95% CI)** |  |
| 3 months | 6.6 (19.2) | 8.1(11.3) | 1.07 (0.79-1.45) | 0.649 |
| 12 months | 20.7 (75.4) | 20.5 (37.2) | **1.62 (1.06-2.48)** | **0.025** |
|  |  |  |  |  |
| **Health care appointments^2^** |  |  |  |  |
| Physician 3 months | 1.8 (1.5) | 1.7 (1.5) | **1.14 (1.09-1.19)** | **<0.001** |
| Physician 12 months | 2.5 (2.5) | 2.3 (2.2) | **1.21 (1.16-1.27)** | **<0.001** |
| Physiotherapist 3 months | 0.9 (3.1) | 0.6 (0.8) | **1.4 (1.38-1.52)** | **<0.001** |
| Physiotherapist 12 months | 1.1 (3.9) | 0.8 (0.8) | **1.23 (1.23-1.23)** | **<0.001** |
| Nurse 3 months | 1.6 (2.7) | 1.6 (1.7) | 1.03 (0.99-1.07) | 0.139 |
| Nurse 12 months | 1.7 (3.1) | 2.1 (2.2) | **0.86 (0.83-0.89)** | **<0.001** |
| Secondary health care 3 months | 0.5 (1.7) | 0.4 (0.8) | **2.02 (1.34-3.04)** | **0.001** |
| Secondary health care 12 months | 0.8 (2.9) | 1.3 (2.7) | **0.64 (0.62-0.67)** | **<0.001** |
